# Innate Immune Training Initiates Efferocytosis to Protect against Lung Injury

**DOI:** 10.1002/advs.202308978

**Published:** 2024-01-26

**Authors:** Yoon‐Young Kang, Dong‐Young Kim, Sang‐Yong Lee, Hee‐Joong Kim, Taehawn Kim, Jeong A. Cho, Taewon Lee, Eun Young Choi

**Affiliations:** ^1^ Department of Biomedical Sciences University of Ulsan College of Medicine ASAN Medical Center Seoul 05505 Republic of Korea; ^2^ Department of Microbiology University of Ulsan College of Medicine ASAN Medical Center Seoul 05505 Republic of Korea; ^3^ Division of Applied Mathematical Sciences College of Science and Technology Korea University Sejong 30019 Republic of Korea; ^4^ Present address: Institute for Clinical Chemistry and Laboratory Medicine Faculty of Medicine Technische Universität Dresden 01307 Dresden Germany

**Keywords:** alveolar macrophages, efferocytosis, inflammation, lung injury, trained immunity

## Abstract

Innate immune training involves myelopoiesis, dynamic gene modulation, and functional reprogramming of myeloid cells in response to secondary heterologous challenges. The present study evaluates whether systemic innate immune training can protect tissues from local injury. Systemic pretreatment of mice with β‐glucan, a trained immunity agonist, reduces the mortality rate of mice with bleomycin‐induced lung injury and fibrosis, as well as decreasing collagen deposition in the lungs. β‐Glucan pretreatment induces neutrophil accumulation in the lungs and enhances efferocytosis. Training of mice with β‐glucan results in histone modification in both alveolar macrophages (AMs) and neighboring lung epithelial cells. Training also increases the production of RvD1 and soluble mediators by AMs and efferocytes. Efferocytosis increases trained immunity in AMs by stimulating RvD1 release, thus inducing SIRT1 expression in neighboring lung epithelial cells. Elevated epithelial SIRT1 expression is associated with decreased epithelial cell apoptosis after lung injury, attenuating tissue damage. Further, neutrophil depletion dampens the effects of β‐glucan on macrophage accumulation, epigenetic modification in lung macrophages, epithelial SIRT1 expression, and injury‐mediated fibrosis in the lung. These findings provide mechanistic insights into innate immune training and clues to the potential ability of centrally trained immunity to protect peripheral organs against injury‐mediated disorders.

## Graphical abstract

Innate immune training triggers myeloid cell production and functional reprogramming. β‐glucan can induce immune training, which increases pulmonary neutrophil accumulation and then boosts efferocytosis by alveolar macrophages. Efferocytosis enhances trained immunity through resolvin D1 release, inducing sirtuin 1 expression in epithelial cells for improved damage resistance. β‐glucan training forms a link among neutrophils, macrophages, and epithelial cells, thereby preventing lung fibrosis.

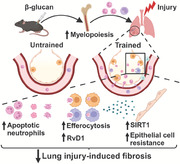

## 1 Introduction

Properly controlled immune cell infiltration is a protective immune response against infection and tissue injury. Immune cells patrol the bloodstream and naïve tissues and promptly respond to an alarm triggered in tissues.^[^ ^1^ ^]^ Both neutrophils and macrophages contribute significantly to acute inflammation and subsequent tissue repair. Neutrophils, which arrive initially, actively remove pathogens and often set the stage for subsequently infiltrating monocytes and macrophages.^[^ ^2^ ^]^ Systemically or locally primed myeloid cells affect distant sites through various transmission mechanisms, including the trafficking of primed cells to the site of insult.^[^ ^3^ ^]^ Myeloid cells that infiltrate into tissues, as well as resident macrophages, actively clear pathogens and apoptotic cells, altering the microenvironment to restore tissue homeostasis. Rapid and efficient efferocytosis (engulfing of apoptotic cells) is critical to tissue repair and homeostasis.^[^ ^4^ ^]^ Efferocytosis per se also enhances macrophage activity in response to tissue damage and infection.^[^ ^5^ ^]^ Efferocytes subsequently secrete anti‐inflammatory and wound‐healing molecules and metabolites, which participate in resolution.^[^ ^6^ ^]^ These soluble molecules and metabolites regulate neighboring stromal cells, such as epithelial cells and fibroblasts, as well as immune cells.^[^ ^7^ ^]^ Failed resolution of acute inflammation results in chronic inflammation. The lungs are constantly or frequently exposed to biological, physical, and chemical stimuli, including bacteria, viruses, radiation, certain medications, and other substances responsible for tissue injury. Abnormal wound healing and tissue regeneration in the lungs result in the initiation and further development of fibrosis.^[^ ^8^ ^]^ Persistent or uncontrolled inflammation and tissue damage in the lung can progress to pulmonary fibrosis (PF). Except for lung transplantation, no effective treatment of PF is currently available.

Innate immune memory is a feature of innate immune cells that acquire memory following an initial encounter and respond better to subsequent, nonspecific stimuli.^[^ ^9^ ^]^ Immune memory confers on cells as either immune training or tolerance following secondary challenges. Unlike tolerance, trained immunity is accompanied by characteristic epigenetic reprogramming, such as histone acetylation at the promoter and regulatory regions of genes involved in the lipid metabolism, cytokine and chemokine production, and phagocytosis.^[^ ^10^ ^]^ Pretreatment with various microbial components, such as β‐glucan, a component of fungal cells, and peptidoglycan, a component of the cell walls of bacteria, or Bacille Calmette‐Guerin (BCG), a whole microorganism vaccine, prior to secondary insult has been found to affect innate immune training.^[^ ^11^ ^]^ Innate immune training induces myelopoiesis and proinflammatory signaling by innate immune cells and their progenitor cells, thereby regulating infection, inflammation, and tumor development.^[^ ^12^ ^]^ Training of innate immune cells with several agonists, such as pathogen‐associated molecular patterns (PAMPs) and damage‐associated molecular patterns (DAMPs), induces metabolic reprogramming in these cells. These metabolites activate epigenetic modifying enzymes to alter the expression of various genes, enhancing innate immune response, such as inflammation and efferocytosis, through immunometabolic circuits.^[^ ^9^, ^13^ ^]^ Moreover, trained immunity has been found to exacerbate inflammatory and autoimmune diseases.^[^ ^13^ ^]^ Development of trained immunity is not limited to mature myeloid cells and their precursors, but also is a feature to other cell types, including dendritic cells, natural killer cells, innate lymphoid cells, stromal and epidermal stem cells, and epithelial cells.^[^ ^9^ ^]^ Increased knowledge of the interplay of trained immune cells with stromal cells allows manipulation of therapeutic approaches to immune‐mediated diseases. The involvement of trained immunity in various inflammatory disorders may be dependent on different cellular and molecular components in immune‐metabolism environments. However, the role of systemically trained immunity in the development of lung injury‐mediated fibrosis remains unclear.

The present study evaluated the role of innate immune training in protecting against bleomycin‐induced lung injury in mice. Myeloid cells systemically trained with β‐glucan, a fungal PAMP, were found to play a role in lung injury. Neutrophil accumulation in the lungs induced macrophage efferocytosis, making neighboring epithelial cells resistant to cellular stress and protecting the lungs against secondary injury. This training of myeloid cells altered lipid metabolism in the microenvironment of lung tissue, thus attenuating lung injury and PF. These findings suggest a substantial link between trained myeloid cells and stromal epithelial cells in protection against tissue damage. Systemically trained immunity may control fibrosis associated with inflammatory, metabolic, and oxidative stresses in the lungs.

## 2 Results

### 2.1 Systemic Trained Immunity Protects Mice against Lung Injury‐Induced Fibrosis

**Figure 1:**
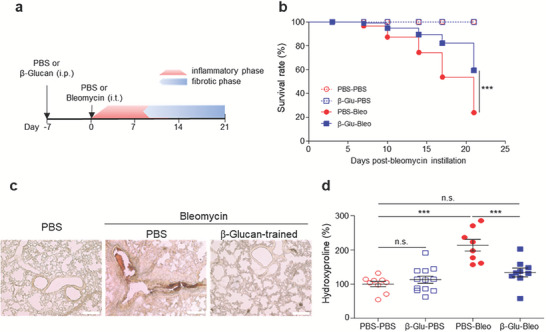
β‐Glucan‐induced trained immunity protects against lung injury‐induced fibrosis. a) Schematic diagram of in vivo training preceded by lung injury. Mice were intraperitoneally administered β‐glucan, followed 7 d later by intratracheal instillation of bleomycin, and monitored for 21 d. b) Survival of PBS‐ or β‐glucan‐trained mice with bleomycin‐induced pulmonary fibrosis. PBS‐PBS *n* = 12 mice, β‐Glu‐PBS; PBS‐Bleo; β‐Glu‐Bleo *n* = 18 mice per group. ***p < 0.0001 by log‐rank test. c) Visualization of collagen deposits in lung sections by sirius red staining. Collagen (red) and muscle fiber (yellow). Scale bar = 100 µm. d) Hydroxyproline content of lung tissues 21 d after bleomycin instillation. *n* = 6–12 mice per group. **p < 0.01; ***p < 0.001 by one‐way ANOVA.

The ability of β‐glucan‐mediated trained immunity to regulate local lung injury‐induced pathologies was assessed using a mouse model of bleomycin‐induced PF. Two overlapping phases have been observed in this model: an earlier inflammatory phase, peaking at 3–7 d post‐bleomycin instillation (dpbi) and gradually declining thereafter, and a subsequent fibrotic phase, with fibrosis initially detected at 10 dpbi and persisting until the end of observation. This model mimics progressive PF with increasing mortality. Treatment of these mice with anti‐inflammatory and anti‐fibrotic agents often results in the resolution of inflammation and tissue repair before the development of fibrosis.^[^ ^14^ ^]^ Mice were intraperitoneally administered PBS or β‐glucan on day −7, followed by intratracheal injection of PBS or bleomycin on day 0 (**Figure** **1**a). Evaluation of mouse mortality during the course of bleomycin‐induced pulmonary fibrosis (BIPF) showed that all mice pretreated with PBS or β‐glucan were alive after intratracheal injection of PBS. Mice pretreated with PBS and subsequently injected intratracheally with bleomycin showed high mortality rates, beginning to die at 7 dpbi with the survival rate gradually decreasing, as expected (Figure 1b). In contrast, mice pretreated with β‐glucan and subsequently treated with bleomycin showed a significantly lower mortality rate (Figure 1b). Deposition of excessive extracellular matrix proteins is a hallmark of fibrosis.^[^ ^15^ ^]^ Collagen deposition was observed in the lungs of PBS (untrained)‐ or β‐glucan‐pretreated (trained) mice at 21 dpbi, with collagen deposition in lung sections being markedly higher in untrained mice than in β‐glucan‐trained mice (Figure 1c). Bleomycin instillation increased the level of hydroxyproline, an indicator of collagen content and fibrosis severity, in the lungs of untrained mice at 21 dpbi, whereas sham instillation did not (Figure 1d). However, systemic β‐glucan pretreatment significantly reduced the levels of pulmonary hydroxyproline at 21 dpbi (Figure 1d). These data indicate that systemic β‐glucan training could protect mice against local BIPF. As prophylactic treatment with β‐glucan is unlikely in a clinical setting, we next asked whether it would be still effective to perform treatment even after lung injury has occurred. After bleomycin instillation, we treated mice at 3 dpbi with β‐glucan or PBS and measured the level of a fibrosis indicator at 37 dpbi and mouse survival rate until 37 dpbi. β‐glucan treatment at 3 dpbi significantly reduced the level of hydroxyproline in the lungs of mice, compared with PBS treatment (Figure S1, Supporting Information). In addition, the survival rate of the mice treated with β‐glucan after lung injury tended to increase compared to the mice treated with PBS, although the difference was not statistically significant (Figure S1, Supporting Information).

### 2.2 Systemic β‐Glucan Pretreatment Induces Neutrophil Infiltration into the Lungs in the Absence of Tissue Injury, Increasing Apoptotic Neutrophils in the Lung

The finding that β‐glucan pretreatment reduced lung injury‐mediated pathology, indicated a need to assess its molecular mechanism. Whole lungs were therefore subjected to single‐cell RNA sequencing (scRNAseq) analyses during the early inflammatory phase (4 dpbi) and the early fibrotic phase (12 dpbi). To determine whether systemic β‐glucan pretreatment influences lung cell populations, whole lungs were dissociated into single cells without removing peripheral blood cells and the cells subjected to scRNAseq analysis. Immune and non‐immune cells were sorted into distinct clusters, with the percentages of distinct types of immune cells in whole lungs including percentages of peripheral blood cells. Systemic β‐glucan pretreatment increased the percentages of neutrophils and monocytes/macrophages but decreased the percentages of T and B lymphocytes in the lungs of trained mice at earlier period (i.e., day 4 after sham instillation, equivalent to day 11 after β‐glucan pretreatment) than in the lungs of untrained mice (Figure S2, Supporting Information), consistent with previous findings.^[^ ^16^ ^]^ In addition, systemic β‐glucan pretreatment increased the percentage of epithelial cells and fibroblasts, even in the absence of lung injury (Figure S2b, Supporting Information), suggesting that systemic β‐glucan pretreatment could affect the lung parenchyma, thereby regulating tissue injury in response to subsequent stimuli.

**Figure 2:**
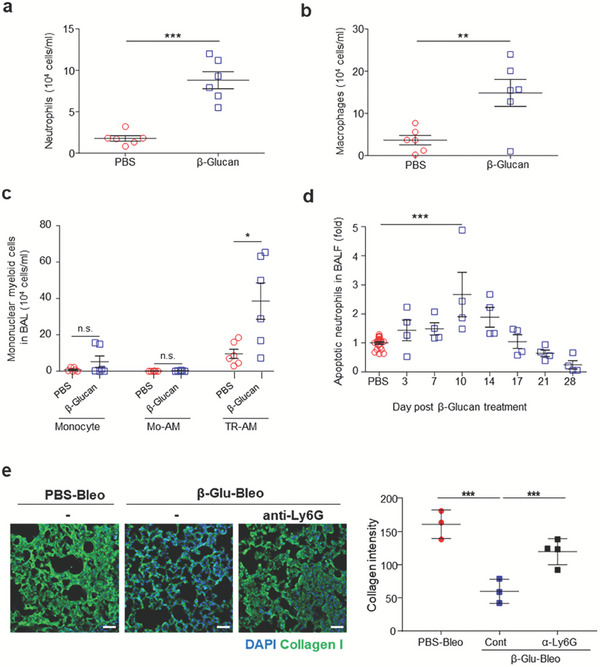
Systemic β‐glucan pretreatment led to accumulation of myeloid cells in the lungs in the absence of tissue injury, increasing apoptotic neutrophils. a,b) Infiltrating and accumulating immune cells in the BALF of untrained and trained mice. Numbers of a) neutrophils (CD11b^+^Gr1^+^) and b) macrophages (F4/80^+^CD45^+^) in BALF 10 d after intraperitoneal injection of PBS (untrained) or β‐glucan (trained). *n* = 6 mice per group. c) Numbers of monocytes (F4/80^−^CD11c^high^SiglecF^−^), monocyte‐derived AMs (Mo‐AM; F4/80^+^CD11c^high^SiglecF^−^), and tissue‐resident AMs (TR‐AM; F4/80^+^CD11c^low^SiglecF^+^) in the BALF of untrained and trained mice 10 d after intraperitoneal injection of PBS or β‐glucan. *n* = 6 mice per group. d) Fold change in numbers of apoptotic neutrophils (AnnexinV^+^Gr1^+^) in the BALF of trained mice 0, 3, 7, 10, 14, 17, 21, and 28 d after intraperitoneal injection of β‐glucan. *n* = 4 mice per group. e) Effect of neutrophil depletion through treatment of mice with anti‐Ly6G mAb on bleomycin‐induced pulmonary fibrosis. Representative images showing collagen type I at 14 dpbi in the lung sections of untrained (PBS) and trained (β‐glucan) mice (left panels). Scale bar = 50 µm. Quantification of the mean fluorescence intensity of collagen (right panels). *n* = 3–4 mice per group. *p < 0.05; **p < 0.01; ***p < 0.001; by two‐sided paired a,b) Student's t‐tests or c–e) one‐way ANOVA.

To determine the mechanism underlying the attenuation of lung injury‐mediated fibrosis in mice systemically pretreated with β‐glucan, changes in immune responses were evaluated in the bronchoalveolar lavage fluid (BALF) of β‐glucan‐trained mice. Even in the absence of bleomycin instillation, the β‐glucan‐trained mice showed higher numbers of myeloid cells, including neutrophils (CD11b^+^Gr1^+^) and macrophages (F4/80^+^CD45^+^), in the BALF 10 d after β‐glucan pretreatment, equivalent to 3 d after sham (PBS) instillation (**Figure** **2**a,b). Analysis of two major alveolar macrophage subpopulations, tissue‐resident alveolar macrophages (TR‐AMs) and infiltrating monocyte‐derived alveolar macrophages (Mo‐AMs) showed that the numbers of TR‐AMs (F4/80^+^CD11c^low^SiglecF^+^‐gated), but not Mo‐AMs (F4/80^+^CD11c^high^SiglecF^−^‐gated), were significantly higher in the lungs of β‐glucan‐trained mice 10 d after β‐glucan pretreatment (Figure 2c). This finding suggested that systemic β‐glucan pretreatment may influence lung‐resident cells, i.e., TR‐AMs, before secondary injury. Neutrophils are short‐lived cells. Of note, the number of apoptotic neutrophils cells in the BALF increased until day 14 after β‐glucan treatment (Figure 2d), suggesting that a certain number of β‐glucan‐trained apoptotic neutrophils are present locally in the lungs prior to tissue injury. Accumulation of neutrophils was observed in other tissues as well, such as intestine and liver, in the trained mice but not in the untrained mice with no neutrophil accumulation (Figure S3, Supporting Information). Neutrophil depletion using an anti‐Ly6G antibody aggravated lung injury‐mediated fibrosis in the mice, as revealed by immunofluorescence staining of collagen type I in lung sections (Figure 2e), indicating that neutrophils play an important role in β‐glucan‐trained immunity. Following bleomycin instillation, however, myeloid cell accumulation in BALF did not differ significantly between untrained and trained mice during the early inflammatory phase, at 3 dpbi (Figure S4, Supporting Information). In the meantime, the concentrations of the cytokines interleukin‐1β (IL‐1β), IL‐6, IL‐4, and IL‐10 in BALF during the course of lung injury‐induced fibrosis were comparable in untrained and trained mice following bleomycin instillation, suggesting that these cytokines are not major contributors to β‐glucan‐attenuated lung injury and fibrosis (Figure S5, Supporting Information). Although myeloid cells accumulated in the lungs of trained mice, the concentrations of proinflammatory cytokines, such as IL‐1β and IL‐6, were very low in the BALF of β‐glucan‐trained mice in the absence of injury, indicating a lack of significant inflammation in peripheral tissue. By contrast, the concentrations of the anti‐inflammatory cytokines IL‐4 and IL‐10 in the BALF of β‐glucan‐pretreated mice increased transiently on days 3 and 10, respectively, following injury (Figure S5, Supporting Information). These findings suggest that a distinct cytokine milieu might partly influence cell phenotypes at several time points in response to a secondary trigger during the course of lung injury. Systemic β‐glucan pretreatment may therefore result in the accumulation of myeloid cells in local tissue before injury, thereby modulating tissue inflammation in response to subsequent local injury.

### 2.3 Systemic β‐Glucan‐Trained Immunity Accelerates Efferocytosis of Alveolar Macrophages

**Figure 3:**
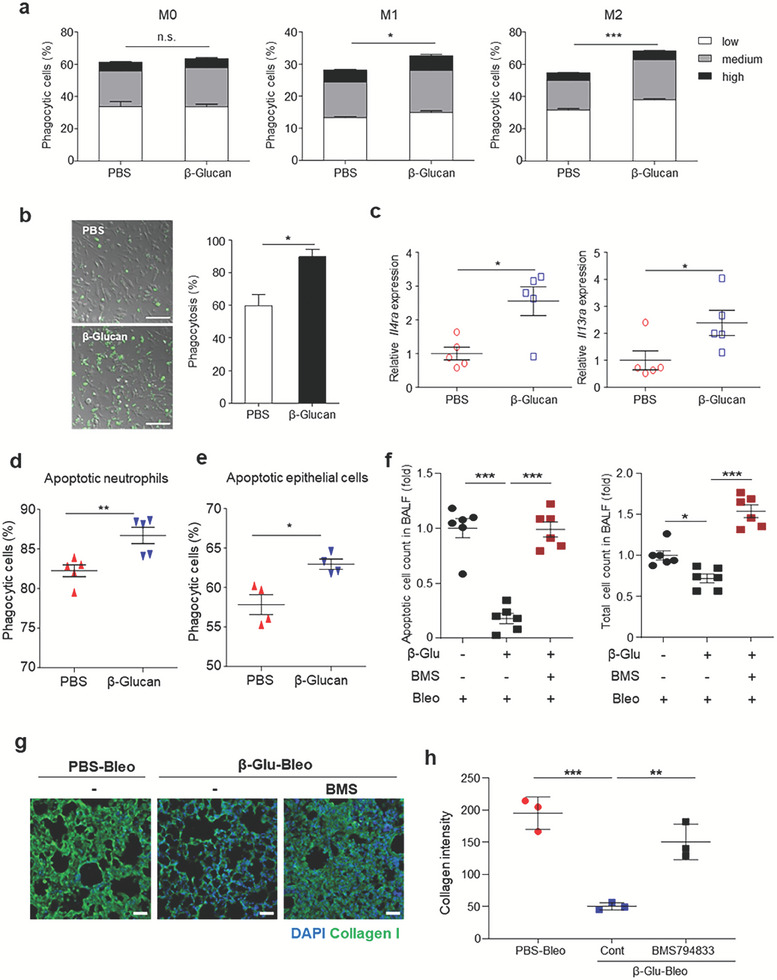
Immune training with β‐glucan accelerates efferocytosis of AMs. a) Phagocytosis of phosphatidylserine‐coated beads by untrained or in vitro‐trained bone marrow‐derived macrophages incubated in the absence (M0‐type) or presence of LPS/IFN‐γ (M1‐type) or IL‐4 (M2‐type). The numbers of cells phagocytosing fluorescent beads were measured by flow cytometry depending on relative fluorescence intensity (low, medium, or high, as indicated in extended data Figure 4). *n* = 5 mice per group. b) Representative confocal microscopy images showing the phagocytic capability of M2 type macrophages cultured as in (a), with phagocytosis quantified. Scale bar = 100 µm. *n* = 3 independent cultures per group. c) Relative expression of the *Il4ra* and *Il13ra* genes in AMs (sorted as SiglecF^+^Gr1^−^) 7 d after intraperitoneal injection of β‐glucan. *n* = 5 mice per group. d,e) Ex vivo efferocytosis of d) apoptotic neutrophils and e) apoptotic epithelial cells by AMs (SiglecF^+^Gr1^−^) isolated from untrained (PBS) and β‐glucan‐trained mice 7 d after β‐glucan pretreatment. *n* = 5 mice per group. f) Relative count of apoptotic cells (left) and total cells (right) in the BALF in the trained mice with lung injury after treatment with a specific efferocytosis blocker, BMS794833. *n* = 6 mice per group. g) Effect of blocking efferocytosis through treatment of mice with BMS794833 on collagen type I production in the lungs of trained mice with lung injury. Representative images of collagen at 14 dpbi in the lung sections of untrained (PBS) and trained (β‐glucan) mice. Scale bar = 50 µm. h) Quantification of the mean fluorescence intensity of collagen (g). *n* = 3–4 mice per group. *p < 0.05; **p < 0.01; ***p < 0.001; by a–f) t‐tests or h) one‐way ANOVA.

Because β‐glucan has been shown to reprogram myeloid cells in bone marrow, and to alter their functional phenotypes,^[^ ^17^ ^]^ the ability of systemic β‐glucan‐training to alter macrophage functional phenotypes was evaluated. To induce in vitro training, bone marrow‐derived macrophages were pretreated with β‐glucan and incubated in the presence of IFN‐γ and LPS, inducing M1‐polarized macrophages, or IL‐4, inducing M2‐polarized (Figure S6a, Supporting Information). The cells were then subjected to phagocytosis of phosphatidyl serine‐coated beads. Both M1‐ and M2‐polarized macrophages, but not M0 macrophages, showed more potent phagocytosis activity upon β‐glucan pretreatment (**Figures** **3**a and S6b, Supporting Information). Moreover, β‐glucan‐pretreated macrophages ingested a higher number of beads, as shown by their stronger fluorescence intensity (Figure 3a,b).

To validate this finding ex vivo, mice were injected intraperitoneally with PBS (untrained) or β‐glucan (trained), and alveolar macrophages were isolated 7 d later. Systemic β‐glucan training of alveolar macrophages was found to significantly increase the expression of genes encoding the receptors *Il4ra* and *Il13ra*, both of which are associated with M2 macrophage polarization, 7 d after β‐glucan pretreatment, suggesting that systemic β‐glucan training may reshape the lungs by suppressing inflammatory responses (Figure 3c). Compared to alveolar macrophages from untrained mice, alveolar macrophages from trained mice showed significantly increased phagocytosis of apoptotic neutrophils and alveolar epithelial cells (Figure 3d,e). To test whether efferocytosis is critical for the β‐glucan‐induced trained immunity‐mediated lung fibrosis prevention, we assessed in vivo efferocytosis in the lungs of β‐glucan‐trained mice. Treatment of mice with β‐glucan decreased early and late apoptotic cells in the lung at 1 dpbi, which was prevented by blocking efferocytosis with a MERTK specific inhibitor, BMS794833 (Figure S7, Supporting Information; Figure 3f), indicating that β‐glucan training enhances in vivo clearance of apoptotic cells by AMs. In addition, β‐glucan‐enhanced efferocytosis reduced the accumulation of total cells in the BALF at 1 dpbi, which was restored by BMS794833 treatment (Figure 3f). Further, inhibition of efferocytosis aggravated collagen production at 14 dpbi in the lungs of β‐glucan‐trained mice with injury (Figure 3g,h). This finding indicates that efferocytosis is required for amelioration of tissue inflammation and fibrosis in the β‐glucan‐trained mice.

**Figure 4:**
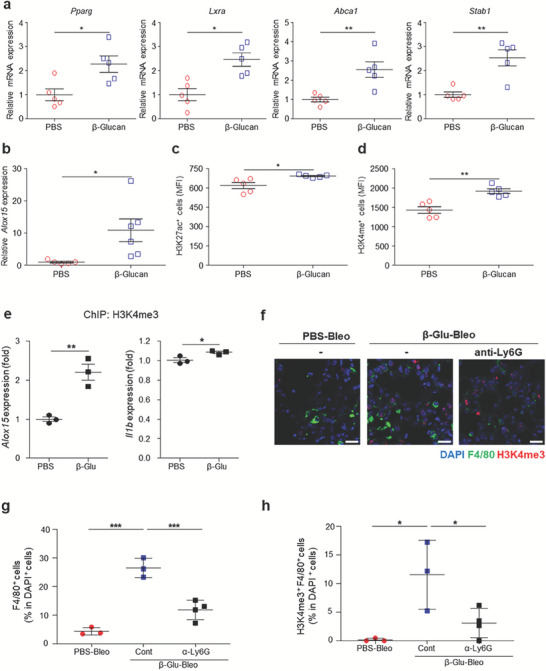
Immune training with β‐glucan increases the transcription of genes involved with phagocytosis and induces epigenetic modification in lung macrophages. a) Relative expression of genes associated with phagocytosis (*Pparg, Lxra, Abca1*, and *Stab1*) in AMs (SiglecF^+^Gr1^−^) isolated from untrained and trained mice 7 d after β‐glucan treatment. *n* = 5 mice per group. b) Relative expression of *Alox15* in whole lungs isolated from untrained (*n* = 5) and β‐glucan (*n* = 6) mice 14 d after β‐glucan treatment. c,d) Global modification of the histones c) H3K27ac and d) H3K4me3 in lung macrophages (F4/80^+^ cells‐sorted) from untrained and trained mice 7 d after PBS or β‐glucan treatment. *n* = 5–6 mice per group. e) ChIP‐qPCR analysis of H3K4me3 enrichment at the *Alox15* and *IL‐1b* locus in lung macrophages (F4/80^+^ cells‐sorted) isolated from untrained and trained mice 7 d after PBS or β‐glucan treatment. One dot represents a pool of lung macrophages isolated from six untrained or trained mice. f–h) Effect of neutrophil depletion through treatment of mice with anti‐Ly6G mAb on the number of macrophages and epigenetic modification in lung macrophages of mice with bleomycin‐induced pulmonary fibrosis. Representative images of H3K4me3 in F4/80^+^ cells at 14 dpbi in the lung sections of untrained (PBS) and trained (β‐glucan) mice (f). Scale bar = 20 µm. Percentages of g) macrophages (F4/80^+^ cells) and h) H3K4me3^+^F4/80^+^ cells in the lung sections (right panel). *n* = 3‐4 mice per group. *p < 0.05; **p < 0.01; ***p < 0.001 by a–e) t‐tests or g,h) one‐way ANOVA.

The increased efferocytosis of alveolar macrophages of trained mice may be due to the upregulated expression of genes involved in the phagocytosis of apoptotic cells, including genes encoding peroxisome proliferator‐activated receptor‐γ (*Pparg*), liver X receptor α (*Lxra*), ATP‐binding cassette transporter 1 (*Abca1*), homeostatic scavenger receptor stabilin‐1 (*Stab1*) (**Figure** **4**a) and others (Figure S8, Supporting Information). Anti‐inflammatory and pro‐resolving genes are upregulated in macrophages following efferocytosis and treatment with the anti‐inflammatory cytokines IL‐4 and IL‐13.^[^ ^18^ ^]^ Indeed, expression of the gene encoding arachidonate 15‐lipoxygenase (*Alox15*), a key enzyme that generates pro‐resolving lipid metabolites,^[^ ^7^, ^18^ ^]^ was significantly higher in the lungs of trained than of untrained mice 14 d after β‐glucan pretreatment (Figure 4b), suggesting that systemic β‐glucan pretreatment facilitates efferocytosis in mouse lungs. Because phenotypic changes in alveolar macrophages of trained mice maybe associated with epigenetic changes, global histone modification was evaluated. The levels of the histones H3K27ac and H3K4me were higher in alveolar macrophages of trained than of untrained mice 7 d after β‐glucan pretreatment (Figure 4c,d), indicating that systemic β‐glucan‐mediated training affects alveolar macrophages, as well as being accompanied by epigenetic modifications and enhanced efferocytosis. Increase of H3K4me and H3K27ac at gene promoters in β‐glucan‐trained macrophages has been reported.^[^ ^10^, ^19^ ^]^ ChIP‐PCR revealed that the increase in H3K4me3 in AMs of the trained mice was associated with increased expression of *Alox15* and *IL‐1b* genes (Figure 4e). These data support the results showing that systemic β‐glucan treatment induces trained immunity in AMs. Meanwhile, injection of mice with anti‐Ly6G dampened the β‐glucan‐mediated increase in H3K4me3 in lung macrophages (Figure 4f,h), indicating that this epigenetic modification requires neutrophils. Of note, neutrophil depletion decreased the number of lung macrophages (Figure 4g). These findings suggest a link between neutrophils and lung macrophages in β‐glucan‐trained immunity.

### 2.4 β‐Glucan‐Enhanced Efferocytosis Enhances the Production of RvD1 and Reduces Tissue Damage Following Lung Injury

**Figure 5:**
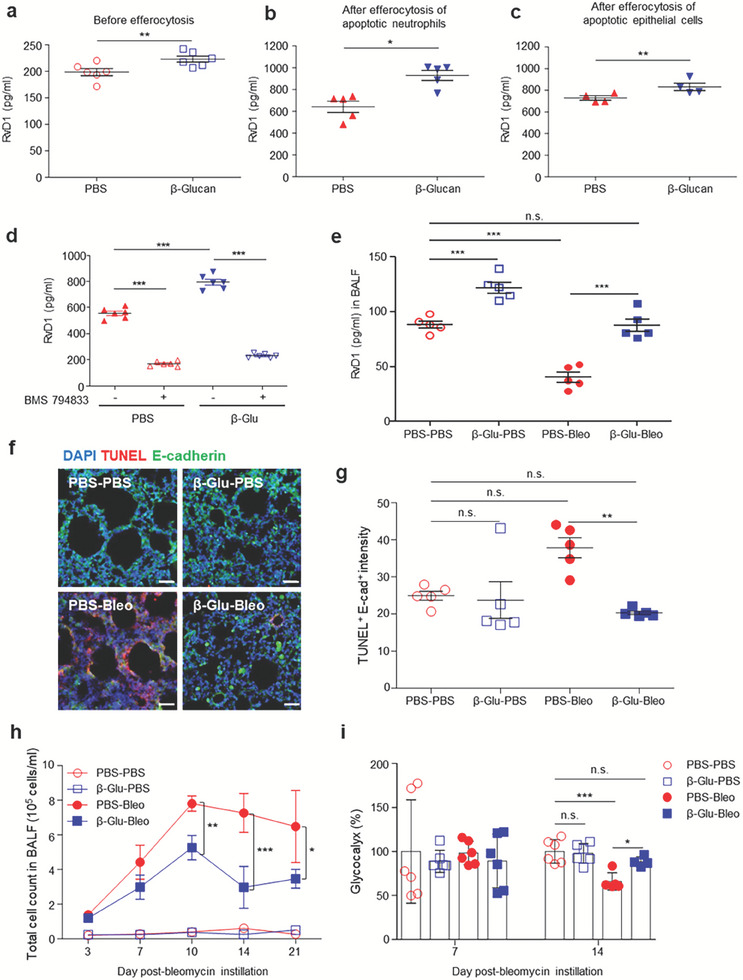
β‐Glucan‐enhanced efferocytosis promotes production of resolvin D1 and reduces tissue damage following lung injury. a–c) Levels of RvD1 in the conditioned media of untrained and a) in vivo‐trained AMs and b,c) efferocytes. Levels of RvD1 in the conditioned media of b) AMs engulfing apoptotic neutrophils and c) AMs engulfing apoptotic lung epithelial cells. *n* = 4–6 per group. d) Effect of blocked efferocytosis on the level of RvD1 in the culture of AMs engulfing apoptotic neutrophils. *n* = 6 per group. e) Levels of RvD1 in the BALF of untrained (PBS) and trained (β‐glucan) mice 10 d after PBS (sham) or bleomycin instillation. *n* = 5 mice per group. f) Representative images showing apoptotic cells (TUNEL) and epithelial cells (E‐cadherin) in the lung sections of untrained (PBS) and trained (β‐glucan) mice 7 d after PBS (sham) or bleomycin instillation. Scale bar = 50 µm. g) Quantification of fluorescence intensity as shown in (e). *n* = 5 mice per group. h) Number of cells in the bronchoalveolar lavage fluid (BALF) of untrained and trained mice following PBS or bleomycin instillation. *n* = 6 mice per group, except for *n* = 5 mice for PBS‐Bleo on day 21. i) Levels of the lung injury marker, glycocalyx, in the lungs of untrained and trained mice 7 and 14 d after bleomycin instillation. *n* = 6 mice per group. *p < 0.05; **p < 0.01; ***p < 0.001 by a–c) t‐tests, d,e,g,i) one‐way ANOVA, and h) two‐way ANOVA.

Sufficient apoptosis is a prerequisite for efferocytosis, which produces resolving lipid mediators and promotes tissue repair.^[^ ^20^ ^]^ In addition, the level of expression of *Alox15* was higher in the lungs of trained than in the lungs of untrained mice. The present study therefore assessed the production of an intrinsic cell‐derived metabolite, RvD1, in these cells. Alveolar macrophages isolated from in vivo β‐glucan‐trained mice produced higher levels of RvD1 than macrophages isolated from untrained mice (**Figure** **5**a). In addition, the concentrations of RvD1 were higher after efferocytosis of either apoptotic neutrophils or epithelial cells than of these cells before additional efferocytosis, as well as being significantly higher in cells trained in vivo with β‐glucan than in untrained cells (Figure 5b,c). Further, specific inhibition of efferocytosis suppressed the release of RvD1, indicating that efferocytosis increases trained immunity in alveolar macrophages (Figure 5d). Further, the levels of RvD1 in the BALF of untrained and β‐glucan‐trained mice with BIPF were assessed. Following lung injury, the RvD1 level decreased in the BALF of untrained mice, yet systemic β‐glucan treatment increased RvD1 in the BALF, the level of which was comparable to that of normal healthy mice (Figure 5e). Of note, the mice with systemic β‐glucan treatment showed increased levels of RvD1 even in the absence of lung injury. The increased efferocytosis and resolving mediator production in the alveolar macrophages of the trained mice suggested that tissue damage may be reduced in the lung parenchyma. Because intratracheal bleomycin instillation first targets epithelial cells,^[^ ^21^ ^]^ leading to subsequent alveolar inflammation, the alveolar epithelium of untrained and trained mice was evaluated following bleomycin instillation. Mice pretreated with β‐glucan showed significantly lower numbers of apoptotic epithelial cells (E‐cadherin^+^ TUNEL^+^) in the alveolar epithelium (Figure 5f,g). Because BIPF is the result of earlier alveolar inflammation,^[^ ^14^ ^]^ the effect of systemic β‐glucan pretreatment on tissue inflammation was evaluated by counting the total numbers of cells in BALF during the course of lung injury. Bleomycin instillation increased the total numbers of both immune and non‐immune cells in BALF during both the inflammatory and fibrotic phases at 3, 7, 10, 14, and 21 dpbi (Figure 5h). However, the increase in the total number of cells in the BALF following bleomycin instillation was significantly lower in β‐glucan‐trained mice than in untrained mice with injury (Figure 5h), indicating that β‐glucan‐training reduces tissue inflammation following injury. Because alveolar epithelial damage leads to pulmonary loss of glycocalyx, a biomarker of tissue injury severity,^[^ ^22^ ^]^ the levels of the remaining glycocalyx in the lung parenchyma were compared. Bleomycin instillation did not alter the level of glycocalyx at 7 dpbi but reduced the levels at 14 dpbi in the lungs of untrained mice (Figure 5i). By contrast, bleomycin had no effect on glycocalyx levels in the lungs of trained mice, even at 14 dpbi (Figure 5i). Taken together, these results suggest that systemic β‐glucan pretreatment resolve further inflammation and may confer resistance to pulmonary epithelial damage.

### 2.5 β‐Glucan‐Trained Macrophages and Efferocytes Induce Epithelial SIRT1 Expression in Lungs, Reducing Apoptosis of Epithelial Cells in Response to Oxidative Cellular Stress

Efferocytosis increases the production of cell‐intrinsic pro‐resolving mediators, limiting tissue damage.^[^ ^23^ ^]^ To identify a pathway responsible for reduced tissue damage in the trained mice, gene expression profiles were evaluated by scRNAseq in epithelial cells and fibroblasts during the later phase (day 12) following tissue injury. Consistent with the attenuated fibrosis observed in the lungs of trained mice, the levels of collagen type I, fibronectin 1, and elastin, all of which are indicators of fibrosis progression,^[^ ^24^ ^]^ were lower in the lung fibroblasts of trained mice than in those of untrained mice (Figure S9, Supporting Information). Lung fibroblasts of trained mice at a later phase after lung injury were found to express lower levels of several genes associated with fibroblast activation, including vimentin (*Vim*), *Cdc42*, *Rac1*, *Rhoa*, *Ki67*, and *Pcna*, than lung fibroblasts of untrained mice (Figure S10a, Supporting Information). By contrast, lung epithelial cells of trained mice showed higher expression of genes associated with tissue homeostasis and repair, such as E‐cadherin (*Cdh1*) and *Hopx*, than lung epithelial cells of untrained mice (Figure S10b, Supporting Information). These findings suggest that alveolar epithelial cells of trained mice undergo efficient repair, whereas lung fibroblasts of untrained mice are activated after tissue injury.

**Figure 6:**
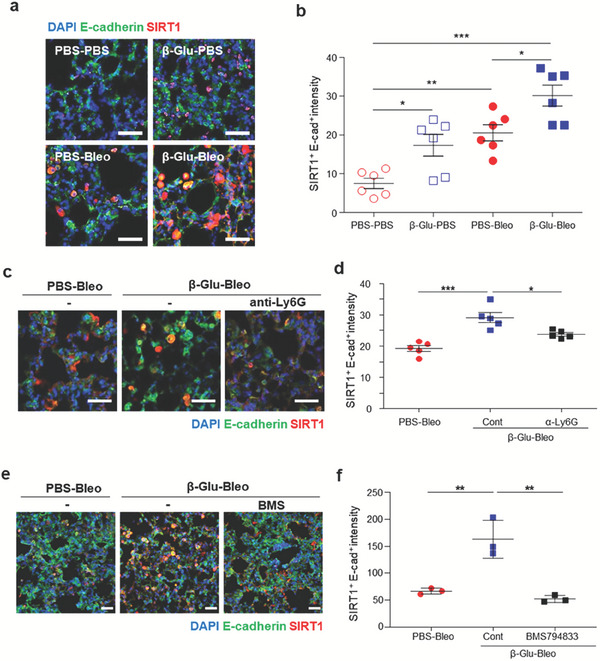
β‐Glucan‐trained immunity induces epithelial SIRT1 expression in the lungs, which requires neutrophil accumulation and macrophage efferocytosis. a) Epithelial SIRT1 protein in the lungs of untrained and trained mice before and after injury. The lungs were isolated 7 d after PBS or bleomycin instillation. Scale bar = 100 µm. b) Quantification of SIRT1^+^ E‐cadherin^+^‐doubly positive cells as in (a). *n* = 6 mice per group. c) Effect of neutrophil depletion through treatment of mice with anti‐Ly6G mAb on epithelial SIRT1 protein levels in the lungs of trained mice with lung injury. Representative images of epithelial SIRT1 at 14 dpbi in the lung sections of untrained (PBS) and trained (β‐glucan) mice (left panels). Scale bar = 100 µm. d) Quantification of SIRT1^+^ E‐cadherin^+^‐doubly positive cells as in (c). *n* = 5 mice per group. e) Effect of blocked efferocytosis through treatment of mice with BMS794833 on the expression of epithelial SIRT1 protein in the lungs of trained mice with lung injury. Representative images of epithelial SIRT1 at 14 dpbi in the lung sections of untrained (PBS) and trained (β‐glucan) mice. Scale bar = 50 µm. f) Quantification of SIRT1^+^ E‐cadherin^+^‐doubly positive cells as in (e). *n* = 3 mice per group. *p < 0.05; **p < 0.01; ***p < 0.001; by one‐way ANOVA.

Efferocytosis may result in the production of pro‐resolving mediators, which target neighboring cells to reprogram metabolism and gene expression in autocrine and paracrine manners.^[^ ^7^ ^]^ Efforts were therefore made to identify resolvin‐targeting molecules that might counteract inflammation and histone acetylation. Lung epithelial cells were less apoptotic in trained than in untrained mice following lung injury, with the lungs of trained mice showing a population of epithelial cells (Figure 5f,g, Figure S10b, Supporting Information). Silent information regulator 2 homolog 1 (SIRT1) is a histone deacetylase positioned at the intersection between epigenetics and metabolism that is involved in immune modulation^[^ ^25^ ^]^ and an epigenetic regulator that adapts the genome to stress. Because SIRT1 is involved in the maintenance of epithelial cell integrity,^[^ ^26^ ^]^ SIRT1 expression in epithelial cells was evaluated in the tissue injury setting using RNAseq. Compared with untrained mice, *Sirt1* was markedly upregulated in the lung epithelial cells of trained mice before and after lung injury (Figure S11a, Supporting Information). The levels of *Sirt1*‐regulated genes involved in anti‐oxidation and anti‐apoptosis, such as *Ncor1*, *Foxo*, *p300*, *Idh2*, and *Aco2*, were higher in lung epithelial cells of trained mice than in those of untrained mice following lung injury (Figure S11b, Supporting Information). To validate the epithelial cell‐specific elevation of *Sirt1* gene expression in trained mice, SIRT1 expression was assessed in lung sections from trained mice. Systemic β‐glucan pretreatment was found to upregulate epithelial SIRT1 expression in the absence of bleomycin instillation (**Figure** **6**a,b). SIRT1 is induced in response to cellular stress.^[^ ^27^ ^]^ Following lung injury, the expression of SIRT1 was higher in the lung epithelial cells (SIRT1^+^ E‐cadherin^+^) of trained mice than in those of untrained mice at 7 dpbi (Figure 6a,b). To test whether β‐glucan‐induced upregulation of epithelial SIRT1 requires neutrophils, neutrophils were depleted using anti‐Ly6G and epithelial SIRT1 expression was assessed in the lungs of mice with lung injury. Depletion of neutrophils significantly reduced the pulmonary epithelial SIRT1 expression in the trained mice (Figure 6c,d), indicating that neutrophils are required for β‐glucan‐induced increase in epithelial SIRT1 expression. Further, in vivo blocking of efferocytosis attenuated the increase in epithelial SIRT1 expression in the lungs of β‐glucan‐trained mice with injury (Figure 6e,f). These findings support a link between neutrophils, AMs, and epithelial cells in β‐glucan‐induced trained immunity.

**Figure 7:**
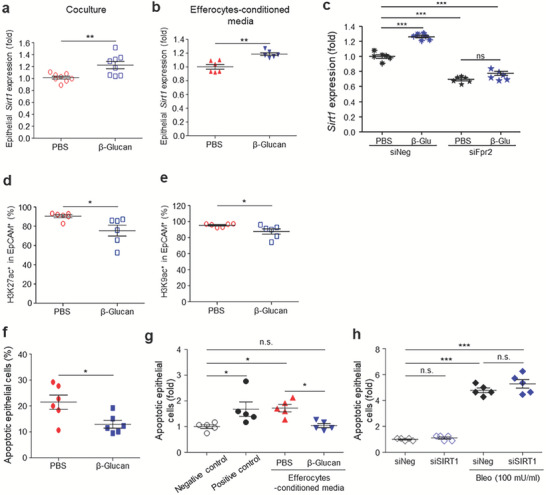
β‐Glucan‐trained macrophages induce epithelial SIRT1 expression, reducing epithelial cell apoptosis upon application of genotoxic and oxidative cellular stress. a) Relative *Sirt1* expression in lung epithelial cells cocultured in transwell with AMs isolated from untrained and in vivo‐trained mice. Epithelial cells were isolated from the lungs of normal untrained mice. *n* = 6 per group. b Relative *Sirt1* expression in lung epithelial cells incubated with the conditioned media of efferocytes. Efferocytes were obtained from coculture of AMs from untrained or trained mice with apoptotic neutrophils. Epithelial cells were isolated from the lungs of normal untrained mice. *n* = 6 per group. c) Effect of knockdown of the RvD1 receptor, Fpr2, on *Sirt1* expression in lung epithelial cells cocultured in transwell plates with AMs isolated from untrained or trained mice. *n* = 6 per group. d,e) Global histone modification (H3K27ac and H3K9ac) in lung epithelial cells (EpCAM^+^ cells) from untrained and trained mice 7 d after β‐glucan pretreatment. *n* = 6 per group. f–h) Apoptosis of lung epithelial cells following bleomycin treatment in vitro. Prior to bleomycin treatment, epithelial cells were incubated with conditioned media of untrained or f) in vivo‐trained AMs or g) efferocytes. Epithelial cells were isolated from the lungs of normal untrained mice. Efferocytes were obtained from coculture of AMs from untrained or trained mice with apoptotic neutrophils. Negative control, none; Positive control, bleomycin but no conditioned media. *n* = 5 per group. h Apoptosis of SIRT1‐knockdown lung epithelial cells following bleomycin treatment in vitro. Epithelial cells isolated from the lungs of normal untrained mice were incubated with conditioned media of in vivo‐trained AMs. *n* = 5 per group. *p < 0.05; **p < 0.01; ***p < 0.001; by a,b,d‐f) t‐tests or c, g,h) one‐way ANOVA

Infiltrating or accumulating trained myeloid cells may affect the lung parenchyma, likely by secreting soluble factors that act on neighboring lung parenchymal cells, inducing epithelial cell resistance to cellular stress, accompanied by epigenetic modifications in epithelial cells. To determine whether β‐glucan‐trained alveolar macrophages induce SIRT1 expression in epithelial cells, normal mouse lung epithelial cells and in vivo β‐glucan‐trained alveolar macrophages were isolated and cultured together. The level of *Sirt1* expression in lung epithelial cells was higher when these cells were cultured in a transwell system with alveolar macrophages from trained than from untrained mice (**Figure** **7**a). Moreover, the conditioned media from efferocytes that further engulfed apoptotic neutrophils increased epithelial *Sirt1* expression in the absence of additional cellular stress (Figure 7b). Upon knockdown of a mouse RvD1 receptor, formyl peptide receptor 2 (*Fpr2*) (Figure S12, Supporting Information), the conditioned media of efferocytes failed to boost epithelial *Sirt1* expression (Figure 7c). These data show that RvD1 promotes epithelial *Sirt1* expression, suggesting the existence of an efferocytosis‐RvD1‐epithelial SIRT1 axis. This increased SIRT1 expression was associated with decreased expression of H3K27ac and H3K9ac in lung epithelial cells (Figure 7d,e). Because SIRT1 may play a role in protecting epithelial cells from cellular stress, the effect of bleomycin treatment on cell apoptosis was evaluated. Apoptosis of epithelial cells was lower when the cells were incubated in the conditioned media of in vivo‐trained than untrained alveolar macrophages (Figure 7f), as well as when the cells were incubated in the conditioned media of efferocytes (Figure 7g). Upon SIRT1 knockdown (verified at the mRNA level; Figure S13, Supporting Information), however, this reduction of epithelial apoptosis was not observed even in the presence of conditioned media from in vivo‐trained macrophages (Figure 7h). These findings indicate that SIRT1 is responsible for epithelial cell resistance to injury, and that trained myeloid cells regulate epithelial SIRT1 induction. By contrast, *Sirt1* expression in alveolar macrophages was lower when trained in vivo with β‐glucan (Figure S14a, Supporting Information), in agreement with results showing that SIRT1 activation had a minimal effect on the production of proinflammatory cytokines by trained monocytes.^[^ ^28^ ^]^ Further analysis showed that macrophage SIRT1 was lower in the lungs of trained mice, than in those of untrained mice following injury (Figure S14b, Supporting Information). Taken together, these findings indicate that a soluble mediator derived from trained alveolar macrophages induces epithelial SIRT1 expression, resulting in reduction of epithelial apoptosis upon subsequent injury.

### 2.6 Systemic β‐Glucan‐Trained Immunity Promotes Pulmonary Production of Pro‐Resolving and Anti‐Inflammatory Lipid Mediators Following Injury

**Figure 8:**
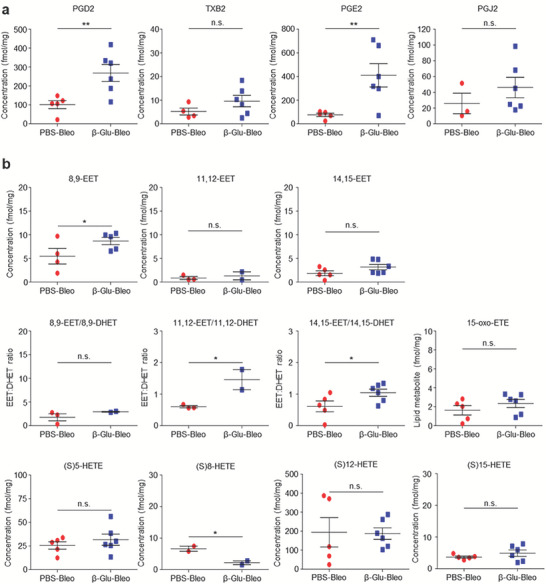
Systemic β‐glucan‐trained immunity promotes production of proresolving and anti‐inflammatory lipid mediators in the lungs. a,b) Levels of endogenous lipid mediators in lung tissues collected a) 7 and b) 14 d after PBS or bleomycin instillation into untrained and trained mice. Lipidomics were performed with the lungs of six mice per group, but some missing data are not shown because the corresponding lipid mediators were not detected. *p < 0.05; **p < 0.01; ***p < 0.001 by t‐tests.

**Figure 9:**
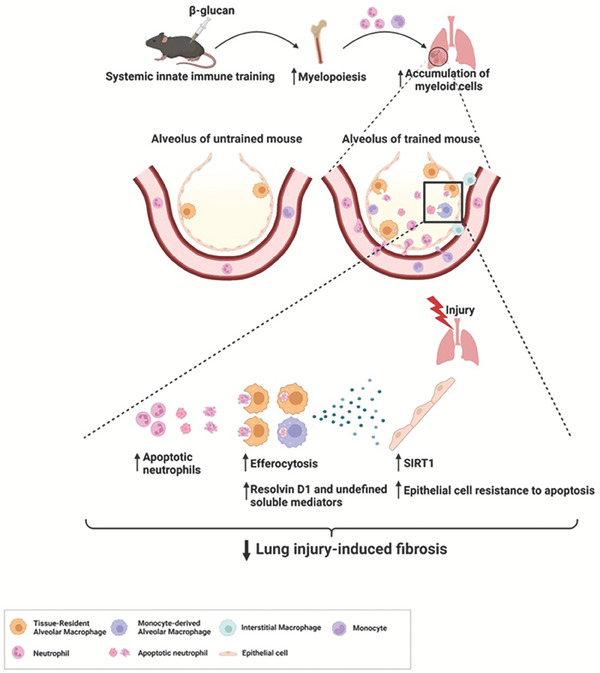
Schematic diagram illustrating the mechanism of systemic β‐glucan‐trained immunity to attenuate lung injury. Created with BioRender.com.

β‐Glucan‐mediated trained immunity involves metabolic reprogramming in response to secondary triggers, potentially favoring tissue repair or resolution, with metabolism regulating tissue injury and fibrosis.^[^ ^29^ ^]^ In addition, β‐glucan‐trained immunity is accompanied by changes in lipid, which may affect phenotypic changes in vivo.^[^ ^30^ ^]^ Efforts were therefore made to identify lipid metabolites that might be generated in the course of injury‐mediated tissue repair or fibrosis in mice. The metabolic milieu in the lungs of untrained and trained mice after lung injury were therefore evaluated by liquid chromatography‐mass spectrometry (LC/MS)‐based lipidomic analysis. Eicosanoids, molecules derived from the oxidation of arachidonic acids (polyunsaturated fatty acids), including resolvins, have been implicated in maintaining tissue homeostasis and fibrosis development.^[^ ^31^ ^]^ Transient but significant increases in some eicosanoids, such as prostaglandin D2 (PGD2) and prostaglandin E2 (PGE2), were observed in β‐glucan‐trained mice during the inflammatory phase 7 d after bleomycin instillation (**Figure** **8**a). By contrast, decreased levels of several eicosanoid metabolites, such as 8,9‐epoxyeicosatrienoic acid (EET), 11,12‐EET and 14,15‐EET, have been observed in the lungs of patients with idiopathic PF.^[^ ^31^ ^]^ During the early fibrotic phase following lung injury 14 d after bleomycin instillation, the levels of 8,9‐EET and the ratio of 14,15‐EET to its metabolic product 14,15‐dihydroxyeicosatrienoic acid (DHET) were higher in the lungs of the β‐glucan‐trained mice than in those of untrained mice with lung injury (Figure 8b). These results reflected the β‐glucan‐mediated alteration of the metabolic milieu in the local tissue. Taken together, these results demonstrate that systemic β‐glucan pretreatment enhanced accumulation of trained myeloid cells in local tissue and increased alveolar macrophage efferocytosis. This resulted in the production of endogenous anti‐inflammatory and proresolving lipid mediators, thus strengthening the integrity of parenchyma cells responding to subsequent injury. This mechanism is schematically illustrated in **Figure** **9**.

## 3 Discussion

The results of this study showed that systemic training with β‐glucan induces accumulation in the lungs of short‐lived neutrophils, which subsequently undergo apoptosis in the lungs. This in turn enhances the efferocytosis of alveolar macrophages, providing a survival signal to epithelial cells, rendering them resistant to cell damage. Thus, systemic β‐glucan training generates a functional linkage of neutrophils, macrophages, and epithelial cells in a peripheral organ.

BCG has been shown to exacerbate systemic sclerosis in mice.^[^ ^32^ ^]^ Trained macrophages activated T and B cells and secreted excess cytokines to induce fibroblast differentiation.^[^ ^32^ ^]^ Repeated injection of low‐grade LPS into mice, however, induced immune tolerance, resulting in reduced production of proinflammatory cytokines by macrophages, thereby limiting inflammation and eventually fibrosis.^[^ ^32^ ^]^ In the experimental model described in this study, systemic β‐glucan‐mediated training resulted in accumulation of myeloid cells in the lungs without skewing to a proinflammatory milieu in the absence of a secondary injury. Moreover, following a single intratracheal bleomycin instillation, β‐glucan training reduced the subsequent accumulation of cells in BALF during the course of injury‐induced fibrosis. Cytokine profiles did not show a bias toward pro‐inflammatory conditions in the lungs of β‐glucan‐pretreated mice during the course of lung injury. Furthermore, systemic β‐glucan treatment in vivo upregulated the expression of IL‐4R/IL‐13R, which favors an anti‐inflammatory (M2) phenotype, in alveolar macrophages. Meanwhile, our study demonstrates that β‐glucan induces anti‐inflammatory and pro‐resolving phenotypes to reduce inflammation and promote lung protection. By contrast, Moorlag et al. showed that β‐glucan protects against *M. tuberculosis* infection in an IL‐1‐dependent manner.^[^ ^33^ ^]^ This discrepancy may be accounted for by disease‐specific differences in the effects of β‐glucan. The type (infectious agent versus sterile inflammation) or magnitude of secondary assaults may determine the extent to which various cells respond to β‐glucan. In addition, source and dosage of β‐glucan may affect β‐glucan‐mediated trained immunity. Immune training may be altered by the types, doses, and routes of administration of PAMPs and DAMPs into mice, as well as altering responses to a secondary attack, thus attenuating or exacerbating inflammation and tissue injury.

Although β‐glucan has been reported to induce proinflammatory responses and promote M1 polarization,^[^ ^34^ ^]^ β‐glucan pretreatment might induce weak inflammation in distant organs. Following a subsequent insult, stimulating tissue inflammation in the tissue, the microenvironment might limit further inflammation. A shift in macrophage phenotype has been shown to be necessary to restore tissue repair and homeostasis.^[^ ^35^ ^]^ A shift from the M1 phenotype to M2 phenotype might be beneficial for β‐glucan trained mice during the course of injury. M2 macrophages have higher efferocytosis ability than M1 macrophages.^[^ ^36^ ^]^ Following β‐glucan treatment, macrophages may be polarized either to the M1 or M2 type, depending on the tissue microenvironment, with significant alterations in efferocytosis. Thus, systemic β‐glucan training may be beneficial for either M1 or M2 polarization milieu in the lungs. Further studies are needed to investigate macrophage plasticity in the lungs of β‐glucan‐trained mice during the development of lung injury‐mediated fibrosis.

Macrophage function may be enhanced by efferocytosis following the influx of neutrophils.^[^ ^37^ ^]^ The collaboration between the two major types of effector cells in β‐glucan‐mediated trained immunity, bone marrow‐derived neutrophils and monocytes/macrophages, may be more efficient than either alone in mitigating tissue damage. Following the efferocytosis of apoptotic neutrophils by trained macrophages, the production of RvD1 was elevated. However, the response of alveolar macrophages to β‐glucan may be improved independently of neutrophils. Indeed, alveolar macrophages trained with β‐glucan in the absence of neutrophils also induced SIRT1 expression by epithelial cells. These alveolar macrophages, however, may have experienced efferocytosis in the trained mouse lungs prior to isolation and culture. Adoptive transfer of trained macrophages to mice in the absence of neutrophils may show whether β‐glucan‐stimulated tissue‐resident macrophages can attenuate tissue damage. Additionally, studies are needed to determine whether metabolites from trained neutrophils alone can modulate SIRT1 induction in epithelial cells.

In addition to bone marrow‐derived myeloid cells, tissue‐resident macrophages can be stimulated directly by β‐glucan. For example, exposure of mice preadministered intranasal gamma herpes virus to a subsequent allergen reduced asthma severity, due to the activity of resident alveolar macrophages.^[^ ^38^ ^]^ In addition, infection with respiratory viruses can result in innate immune memory in alveolar macrophages with the help of CD8 T cells, but not with bone marrow progenitors.^[^ ^39^ ^]^ Various macrophage subpopulations are thought to be specifically involved in the course of lung tissue damage after β‐glucan pretreatment. Systemic pretreatment with β‐glucan increased the number of alveolar macrophages (F4/80^+^CD45^+^), with innate immune training accompanying central myelopoiesis thought to be responsible for the accumulation of both alveolar macrophages and neutrophils in lung tissue, perhaps accompanied by cell infiltration into other tissues or organs. Indeed, peritoneal injection of β‐glucan led to accumulation of myeloid cells in the intestine and liver as well, which is in line with a recent report.^[^ ^40^ ^]^ Although TR‐AMs are relatively hyporesponsive compared with Mo‐AMs, reactivity is dependent on the tissue environment. It is unclear whether β‐glucan treatment affects the self‐renewal of TR‐AMs within a short period of time by responding to cytokines, such as GM‐CSF, TGF‐β, and IL‐10, produced by the TR‐AMs or surrounding cells.^[^ ^41^, ^42^ ^]^ One limitation of the present study was that it did not strictly distinguish between TR‐AMs and Mo‐AMs as the main players of efferocytosis in vivo in β‐glucan‐trained mice (e.g., using lineage tracing and combinations of distinct subsets‐specific markers). This study did not validate whether systemic β‐glucan acted directly on TR‐AMs in the lungs, or whether the function of TR‐AMs was enhanced by β‐glucan treatment‐induced changes in the tissue microenvironment. In addition, Mo‐AMs can be converted to TR‐AMs after several weeks, and the numbers of TR‐AMs decrease following tissue damage.^[^ ^43^ ^]^ Therefore, β‐glucan‐mediated myelopoiesis of monocytes may contribute to the TR‐AM pool. In either case, the β‐glucan‐triggered increase in neutrophils initiated efferocytosis by AM. In the present experimental setting, Mo‐AMs may not play an active role in the attenuation of lung injury. Mo‐AMs may be responsible for a cytokine storm early in tissue damage and later contribute to fibrosis.^[^ ^44^ ^]^ Because these β‐glucan‐pretreated mice did not experience further lung inflammation or serious fibrosis, TR‐AMs likely contributed more to tissue protection than Mo‐AMs. Alternatively, Mo‐AMs may experience phenotypic plasticity, similar to that of TR‐AMs, in response to the surrounding environment.^[^ ^41^ ^]^ Meanwhile, a recent study showed that β‐glucan elicits lung interstitial macrophage training and that lung interstitial macrophages play a crucial role in PF.^[^ ^40^, ^45^ ^]^ In our single cell RNAseq analysis of whole lungs of untrained and β‐glucan‐trained mice, the relative number of interstitial macrophages (F4/80, CD11b, CX3CR1; CX3CR1, CCR2, MafB, MHCII [H2‐Eb1, H2‐Aa, and H2‐Ab1]) in the lungs of trained mice underwent dynamic alterations during the course of lung injury (data not shown). On day 4 after the sham/bleomycin instillation, there was an increase in lung interstitial macrophages of trained mouse in the absence of injury. However, following injury, this population decreased, with no discernible difference between untrained and trained mice. On the other hand, on day 12 after sham/bleomycin instillation, there was a rise in the interstitial macrophage population, particularly noticeable in the lung of trained mouse. As such, it is tempting to speculate that interstitial macrophages play a limited role in the efferocytosis of early arriving neutrophils in our model. Nevertheless, given the dynamic plasticity of macrophages and the transition between distinct subsets of macrophages in the lung under abnormal conditions, we cannot rule out the possibility that lung interstitial macrophages may be involved, to some extent, in β‐glucan‐mediated training during the course of lung injury.

β‐Glucan‐mediated training inhibited the influx of excessive inflammatory cells into the tissue parenchyma upon injury. Furthermore, despite tissue injury, the number of bone marrow cells did not increase further in trained mice (data not shown), indicating that additional new myelopoiesis is not necessary for β‐glucan‐mediated training as tissue injury progressed. As a result, β‐glucan‐mediated training did not amplify inflammation after tissue injury. Continued proliferation of myeloid cells during an increased inflammatory response may elicit adverse effects of training immune response.^[^ ^12a^ ^]^ Upon their engulfment of neutrophils, tissue‐resident macrophages suppress the expression of proinflammatory cytokines while inhibiting granulopoiesis.^[^ ^46^ ^]^ Myeloid cells accumulating in the peripheral tissues of trained subjects might affect the parenchyma. The present study found that β‐glucan‐mediated training upregulates SIRT1 expression in lung epithelial cells and reduces epithelial apoptosis caused by tissue damage. SIRT1 has been reported to inhibit the apoptosis of various cells, including lung epithelial cells, exerting a beneficial effect in vivo, results consistent with the present findings.^[^ ^47^ ^]^ Furthermore, SIRT1 expression was found to be reduced in the lungs of patients with idiopathic PF, with upregulation of SIRT1 expression shown to promote the regenerative capacity of alveolar type 2 cells and alleviate PF.^[^ ^48^ ^]^ The present study found that epithelial cells underwent apoptosis upon exposure to bleomycin in vitro, and that this apoptosis was significantly suppressed by the conditioned media of trained macrophages, indicating that trained macrophages maintain the viability of stromal cells.

Macrophage‐derived RvD1 has been proposed as a potential inducer of SIRT1 expression in epithelial cells.^[^ ^49^ ^]^ The present study showed that β‐glucan training increased resolvin in macrophage culture medium, with the conditioned media of trained macrophages increasing epithelial SIRT1 induction and reducing apoptotic epithelial cells. Moreover, transient but significant increases in anti‐inflammatory and resolving eicosanoids, such as PGD_2_ and PGE_2_, were observed in the lung exudates of β‐glucan‐trained mice during the inflammatory phase of the model following lung injury. Although initial excess of prostaglandins and leukotrienes contribute to chronic inflammation, PGE_2_ and PGD_2_ stimulate anti‐inflammatory circuits. PGD_2_ stimulates macrophages to produce IL‐10, whereas PGD_2_ can be converted to PGJ2 and 15‐dPGJ2 to activate PPARγ, driving resolution.^[^ ^7^ ^]^ By contrast, decreased levels of some eicosanoid metabolites of arachidonic acid are involved in the development of PF.^[^ ^31^ ^]^ These findings were supported by the results of the present study, which showed that levels of 8,9‐EET and 14,15‐EET/14,15‐DHET were significantly higher, and the level of (S)8‐HETE was significantly lower, in the lungs of β‐glucan‐trained mice than in those of untrained mice during the early fibrotic phase following lung injury.

During the preparation of this manuscript, another study reported that parenteral BCG injection trains lung‐resident macrophages, independently of training monocyte‐derived macrophages.^[^ ^50^ ^]^ BCG induced intestinal inflammation and altered the intestinal microbiome, with the resulting microbiome producing distinct microbial metabolites that are transmittable to the lungs and train the lung‐resident macrophages in situ, protecting against subsequent *Mycobacterium tuberculosis* infection. The present study suggests that systemic β‐glucan‐trained myeloid cells migrate homeostatically to the lungs in the absence of insult, establishing a lung microenvironment that better protects against subsequent insult. β‐Glucan‐trained macrophages are involved in the efficient efferocytosis of apoptotic neutrophils and epithelial cells, blocking further inflammation in the lungs and rendering the lungs resistant to tissue injury. Alterations in the production of lipid metabolites also occurred in the lungs of β‐glucan‐trained mice, but inflammation of local tissue was not observed before secondary insult.

As we demonstrated that systemic administration of β‐glucan after mice lung injury significantly decreased the level of hydroxyproline in the lung, we believe β‐glucan could have therapeutic potential against PF in the clinic. However, β‐glucan post‐treatment did not significantly alleviate the mortality of mouse with PF. Adjustments in dosage and frequency of β‐glucan administration could enhance the survival rate significantly.

Taken together, the results of the present study suggest that trained immunity slows or weakens the progression to fibrosis, thus enhancing tissue repair and regeneration. β‐Glucan‐mediated immune training is applicable to the conditions or disorders characterized by poor phagocytosis in tissues, such as aging, smoking, trauma, and infection. In addition, this trained immunity is applicable to injury in other tissues, such as skin, muscle, and the nervous system; epithelial injury caused by genotoxic stresses such as radiation and smoking; and tissue repair disorders such as aging and diabetes.

## 4 Experimental Section

### Ethics Statement

All animal studies were approved by the Institutional Animal Care and Use Committee of the ASAN Institute for Life Science (Project number: 2021‐12‐296).

### Mouse Model of In Vivo β‐Glucan Training Followed by Bleomycin‐Induced Lung Injury

All mice were maintained under specific‐pathogen free conditions. Six‐week‐old male C57BL/6 mice were purchased from Orient Bio (Seongnam, Korea) and allowed to adapt for at least 1 week prior to experiments. Mice were randomly assigned to control and treatment groups. Mice were intraperitoneally injected with β‐glucan peptide from the fungus *Trametes versicolor* (25 mg kg^−1^; InvivoGen, CA, USA) or PBS. The dose of β‐glucan titrated yielded consistent results. After 7 d, the mice were subjected to bleomycin‐induced lung injury. Under anesthesia with Avertin solution (Sigma), the trachea of each mouse was surgically exposed, followed by intratracheal administration of bleomycin (3 U kg^−1^; Sigma, B5507) in 40 µL PBS or PBS alone. The mice were sacrificed as indicated, and their BALF, lungs, and bone marrow tissues were further examined.

### β‐Glucan Treatment of Mice after Bleomycin‐Induced Lung Injury

Mice were intratracheally instilled with bleomycin (3 U kg^−1^; Sigma). After 3 d, the mice were intraperitoneally injected with β‐glucan peptide (25 mg kg^−1^; InvivoGen) or PBS. The survival rates of the mice were monitored until 37 dpbi. Mice were sacrificed at 37 dpbi and the lungs were subjected to the hydroxyproline assay.

### Collagen and Hydroxyproline Assessment of Fibrotic Lungs

Collagen deposition in the lungs was visualized using a Picro Sirius Red staining kit (Abcam). Mice were transcardially perfused with PBS; their lungs were removed, fixed with 4% paraformaldehyde, immersed in sucrose solution, and embedded in OCT compound. Cryostat sections of 15 µm were prepared. The frozen sections were air‐dried, washed three times with PBS (for 5 min each), and stained with Sirius Red. The images were taken by a DM IL microscope (Leica). Hydroxyproline in lungs was measured using a hydroxyproline colorimetric assay kit (BioVision). In brief, lungs were homogenized in 1 mL distilled water and hydrolyzed with 12 m HCl at 120 °C for 3 h. Then, 10 µL aliquots were added to each well of a 96‐well plate, and the solutions were evaporated under vacuum. The samples were incubated with Chloramine T and DMAB solutions for 1.5 h at 60 °C, and absorbance at 560 nm was determined using a microplate reader (Synergy HT, BioTek, Winooski, VT, USA).

### Analysis of Bronchoalveolar Lavage Fluid (BALF)

BALF was collected by injecting 1 mL PBS into the trachea of each mouse, and the solutions were centrifuged at 800 x *g* for 5 min at 4 °C. Each supernatant was transferred to a fresh tube for cytokine analysis, whereas each cell pellet was used for cell counting and immunotyping. To identify cell populations, the cells were incubated with antibodies against CD11b (clone: M1/70, BioLegend), Gr1 (clone: RB6‐8C5, BioLegend), F4/80 (clone: BM8 BioLegend), and CD45 (clone: 30‐F11, BioLegend) and subjected to flow cytometry (BD Accuri C6). Apoptotic cells were detected using a FITC Annexin V Apoptosis detection kit (catalog no. 556 547, BD Biosciences). Cytokine concentrations in BALF were measured by conventional ELISA using uncoated ELISA Kits (ThermoFisher Scientific) for mouse IL‐1β (catalog no. 88‐7013‐88), IL‐6 (catalog no. 887064–88), IL‐4 (catalog no. BMS613), and IL‐10 (catalog no. 88‐7105‐88).

### Immunohistochemistry

Following transcardial perfusion with PBS, the lungs were fixed with 4% PFA overnight at 4 °C, followed by sequential incubation in 15% and 30% sucrose solutions. The lungs were embedded in OCT and sliced into 10 µm thick sections. These sections were post‐fixed and permeabilized for 10 min with 0.1% Triton X‐100 in PBS. The slides were rinsed with PBS and incubated with blocking buffer (10% normal goat serum, 5% BSA in PBS) for 1 h at room temperature, followed by incubation with each primary antibody overnight at 4°C. The primary antibodies and their dilutions were as follows: anti‐F4/80 (clone: BM8, catalog no. 123110, BioLegend, 1:50), anti‐E‐cadherin (clone: 24E10, catalog no. 3195S, Cell Signaling Technology, 1:1000), anti‐SIRT1 (clone: 19A7AB4, catalog no. ab110304, Abcam, 1:100), lectin‐FITC (catalog no. L4895, Sigma, 1:100), anti‐collagen I (catalog no. NB600‐408, Novus Biologicals, 1:300), and anti‐H3K4me3 (catalog no. PA5‐27029, Thermo Fisher Scientific, 1:200). After washing with PBS, the sections were incubated with the appropriate secondary antibodies for 1 h at room temperature. The sections were washed with PBS and incubated with DAPI solution (1 µg mL^−1^) for 10 min at room temperature, followed by the addition of Fluoromount solution (Electron Microscopy Sciences, Hatfield, PA, USA) and analysis by confocal microscopy (LSM 710; Zeiss, Oberkochen, Germany). Fluorescence intensity was measured using ZEN 2.6 software. Apoptosis in the lung sections was measured using annexin V apoptosis detection kits (catalog no. 556547, BD Biosciences) or TUNEL assay kits (catalog no. ab66110, abcam).

### Depletion of Neutrophils

Mice were intraperitoneally injected with anti‐mouse Ly6G (0.25 mg/head/dose, InVivoMAb, catalog no. BE0075‐1) at one day (at −8 dpbi) before β‐glucan treatment (at −7 dpbi) (25 mg kg^−1^, intraperitoneal injection), and repeatedly injected three times per week until sacrificed, followed by bleomycin instillation (at 0 dpbi) (3 U kg^−1^, intratracheal injection). The mice were sacrificed at 14 dpbi and the lungs were subjected to immunohistochemistry. Quantification was performed by counting the stained cells or measuring fluorescence intensity after taking images at 200x magnification. The average cell count or the mean fluorescence intensity were estimated in 4–5 randomly selected squares of respective section images.

### In Vitro Phagocytosis

To obtain in vitro‐trained macrophages, mouse bone marrow cells were isolated and cultured in the presence of M‐CSF (315‐02, Peprotech; 20 ng mL^−1^). On day 5, the bone marrow‐derived macrophages (BMDMs) were seeded at 5 × 10^5^ cells per well in six well plates, and the cells were incubated overnight. An aliquot of β‐glucan (Invivogen; 10 µg mL^−1^) was added to each well, followed by incubation for 24 h. The conditioned media were removed and replaced with fresh media, with culture continued for 3 d. For phagocytosis assay, β‐glucan‐trained and untrained BMDMs were stimulated with IFN‐γ/LPS or IL‐4 for 24 h, followed by incubation for 45 min with beads coated with phosphatidylcholine:phosphatidylserine:phosphatidylserine‐FITC at a ratio of 8:2:0.5).^[^ ^51^ ^]^ The fluorescence of beads in cells was measured by flow cytometry (BD) and confocal microscopy (Zeiss).

### Ex Vivo Efferocytosis

Mice trained in vivo by injection of β‐glucan (25 mg kg^−1^) and untrained mice, as above, were sacrificed after 7 d. Their lung tissues were minced and digested with collagenase I (17104‐017, Gibco) and Dispase (17105‐041, Gibco) for 1 h at 37 °C, and the cells were filtered through a cell strainer. To remove erythrocytes, single cells were incubated with RBC lysis buffer. To obtain AMs, single‐cell suspensions were pelleted, and resuspended in sorting buffer (1% BSA, 0.5 × 10^−3^ m EDTA in HBSS) and incubated with antibodies against Siglec F (catalog no. MAB17061‐100, R&D Systems) and Gr1 (Clone: RB6‐8C5, catalog no. 553122, BD Biosciences). The Siglec F^+^/Gr1^−^ cells were sorted by a flow cytometry (FACS Aria SORP, Becton Dickinson and Company). To prepare apoptotic neutrophils and epithelial cells, neutrophils were isolated from bone marrow cells, as described previously.^[^ ^52^ ^]^ Naïve lung epithelial cells were isolated from normal BL6 mice. Freshly isolated single‐cell suspensions were incubated in epithelial cell growth basal media (catalog no. CC‐3119, Lonza) for 5 d at 37 °C. The purity (greater than 80%) of epithelial cells was assessed by flow cytometry using anti‐E‐cadherin antibody (clone: 24E10, catalog no. 3195, Cell Signaling Technology). Neutrophils and epithelial cells were triggered with hydrogen peroxide (9.8 × 10^−3^ m) and incubated for 48 h to induce apoptosis, with 99.5% of neutrophils and 80.6% of epithelial cells being apoptotic, as assessed by flow cytometry using annexin V‐FITC (catalog no. 556547, BD Biosciences). Independently, the apoptotic neutrophils and epithelial cells were stained with CFSE (5 × 10^−3^ m, 15 min, 37 °C). Ex vivo efferocytosis was performed by incubating AMs with apoptotic neutrophils or lung epithelial cells at a ratio of 1:2 for 2 h at 37 °C, with the fluorescence intensity measured by flow cytometry.

### In Vivo Efferocytosis and Inhibition of Efferocytosis

Mice were untrained or trained with β‐glucan, followed by bleomycin instillation after 7 d. At 1 dpbi, BALF was collected and apoptotic cells were analyzed using annexin V and propidium iodide. To inhibit in vivo efferocytosis, mice were untrained or trained at −7 dpbi and then mice were intraperitoneally injected with BMS794833 (25 mg kg^−1^; catalog no. 26180, Cayman Chemical, Ann Arbor, MI) 30 min prior to bleomycin instillation at 0 dpbi. On the indicated days post bleomycin instillation, BALF or lung tissues were collected for further analyses. In some experiments, to determine the effect of ex vivo efferocytosis by trained macrophages, sorted AMs (1 × 10^5^ cells per well) were plated and treated with BMS794833 (at a concentration of 10 × 10^−6^ m) 1 h before adding apoptotic neutrophils (5 × 10^5^ cells per well), and incubated for 24 h at 37 °C. Their supernatants were collected for assessment of RvD1 by ELISA (MBS058806, Mybiosource).

### Assays of Epithelial SIRT1 Expression and Apoptosis in Cultures with Macrophages

To determine the effect of trained macrophages on *Sirt1* expression in lung epithelial cells, primary lung epithelial cells and sorted AMs were cocultured in a transwell for 60 h at 37 °C. Their supernatants were collected, and RvD1 concentrations were measured by ELISA (MBS058806, Mybiosource). Lung epithelial cells were incubated in supernatant, acting as conditioned medium, at 37 °C for 60 h, and *Sirt1* expression in these cells was determined. Briefly, total RNA was isolated from these cells using TRIzol reagent (Invitrogen), and cDNA was synthesized using a reverse transcriptase kit (Promega). The cDNAs were used as a template for amplification by real‐time PCR using LightCycler 480 SYBR Green 1 Master Mix (Roche) and primer sequences for mouse *Sirt1* (forward, 5′‐ACT CCT CAC TAA TGG CTT TCA TTC‐3′; reverse, 5′‐GGT GGA GGA ATT GTT TCT GGT AAT‐3′) and mouse *18S* (forward, 5′‐CGC GGT TCT ATT TTG TTG GT‐3′; reverse, 5′‐AGT CGG CAT CGT TTA TGG TC‐3′). In some experiments, the epithelial cells were incubated in the absence or presence of conditioned medium at 37°C for 60 h, followed by treatment with bleomycin (100 mU mL^−1^) for 12 h at 37 °C. The resulting cells were stained using a FITC Annexin V/Apoptosis detection kit 1 (BD Pharmagen, 556547) and analyzed by flow cytometry.

### Histone H3 Modification Analysis

Mice trained in vivo by injection of β‐glucan (25 mg kg^−1^) and untrained mice, as above, were sacrificed after 7 d. Their lungs were isolated and treated with collagenase and dispase to yield single‐cell suspensions. After lysing RBCs, the cells were fixed with by incubation with CellCover solution (Anacyte Laboratories, 800‐125) for 10 min at room temperature and centrifuged at 500 x *g* for 5 min. After discarding the supernatant, cell fixation and permeabilization solution (Invitrogen, 005523‐00) was added to each pellet, followed by vortexing and incubation for 1 h at room temperature or 4 °C. The cells were washed, and staining buffer (eBioscience Foxp3/Transcription staining buffer set) was added, followed by the addition of antibodies (1:50) and incubation for 30 min at room temperature. These antibodies included anti‐histone H3 (D1H2, 12167S), anti‐tri‐methyl‐histone H3 (K4) (C42E8, 12064S), anti‐acetyl‐histone H3 (Lys 9) (C5B11, 4484S), and anti‐acetyl‐histone H3 (Lys27) (D5E4, 15485S), all from Cell Signaling. Cells were classified as epithelial cells by their expression of EpCAM and as macrophages by their expression of Siglec F. After washing, these cells were subjected to flow cytometry.

### Chromatin Immunoprecipitation‐PCR Assay

Lungs of untrained mice or mice trained with β‐glucan were dissociated and macrophages were sorted using anti‐F4/80. DNA was isolated from the sorted lung macrophages, and subjected to ChromaFlash High‐Sensitivity ChIP Kit analysis (Epigentek, Farmingdale, NY, USA). H3K4me3‐mediated promoter activation was assessed by PCR using the mouse primers Alox15 (forward, 5′‐TAC CTG TGG TTG ATC GGA CA‐3′; reverse, 5′‐TGC CAT TTC TGC ACT CTC AC‐3′) and IL‐1b (forward, 5′‐CTT TCC CGT GGA CCT TCC AG‐3′; reverse, 5′‐ATA TGG GTC CGA CAG CAC GA‐3′).

### Assays for Receptor Expression in AMs and Epithelial Cells

Receptor mRNA expression was evaluated by real‐time PCR using primers for mouse *Il4ra* (forward, 5′‐TCT GCA TCC CGT TGT TTT GC‐3′; reverse, 5′‐GCA CCT GTG CAT CCT GAA TG‐3′) and mouse *Il13ra* (forward, 5′‐AGC CTG GAG AAA AGT CGT CA‐3′; reverse, 5′‐GGC ACC ATT TTT GAG GAG AA‐3′). Expression of genes associated with efferocytosis was evaluated using primers for mouse *Pparg* (forward, 5′‐AAG ATG TAC CCG TCC GTG TC‐3′; reverse, 5′‐TGA AGG CAG GCT CGA GTA AC‐3′), mouse *Lxra* (forward, 5′‐GGA TAG GGT TGG AGT CAG CA‐3′; reverse, 5′‐GGA GCG CCT GTT ACA CTG TT‐3′), mouse *Abca1* (forward, 5′‐CAC CCC TTG AAC CTC ACT AAA CA‐3′; reverse, 5′‐AAA GGA CAT CGC AAA GAT GAC A‐3′), mouse *Stab1* (forward, 5′‐AGG GGA CTC CAA GAA AAC‐3′; reverse, 5′‐CCA CAG TTC TCC AGG ATC‐3′), and mouse *Alox15* (forward, 5′‐CAG GGA TCG GAG TAC ACG TT‐3′; reverse, 5′‐GAT TGT GCC ATC CTT CCA GT‐3′). Internal control gene expression was evaluated using primer for mouse *18S* (forward, 5′‐CGC GGT TCT ATT TTG TTG GT‐3′; reverse, 5′‐AGT CGG CAT CGT TTA TGG TC‐3′).

### Semi‐Quantitative RT‐PCR Assays for Myeloid Cell Accumulation in Tissues

The relative expression in liver, intestine, and spleen of untrained and trained mice was evaluated by real‐time PCR using the mouse primers *Ly6g* (forward, 5′‐TGG ACT CTC ACA GAA GCA AAG‐3′; reverse, 5′‐GCA GAG GTC TTC CTT CCA ACA‐3′) and *Ccr2* (forward, 5′‐ GGT CAT GATC CCT ATG TGG‐3′; reverse, 5′‐ CTG GGC ACC TGA TTT AAA GG‐3′).

### siRNA Transfection

Mouse primary epithelial cells were seeded in 12‐well culture plates (1 × 10^5^ cells per well) and transfected with 10 × 10^−6^ m mouse *Sirt1* siRNA (Santacruz) or *Fpr2* siRNA (MyBioSource, MBS8237316) in serum‐free opti‐MEM media using lipofectamine reagent (Invitrogen, 13778‐100). After incubation for 4 h, the medium was replaced with fresh medium, and the cells were further incubated for 24 h. Nonspecific siRNA was used as a negative control (siNeg).

### scRNAseq Using Whole Lung Tissues

Mouse lungs were collected, minced, and digested with collagenase I (Gibco) and Dispase (Gibco) for 1 h at 37 °C. Single cells were filtered through a 40 µm cell strainer, RBCs were lysed, and the viability of the remaining cells was tested. Samples with >90% cell viability were used for single‐cell RNAseq, which was performed at ebiogen Inc. (Seoul, Korea). Briefly, single‐cell RNAseq libraries were prepared using 10× Chromium next gem single cell 3′ reagent kits v3.1. All libraries were pooled and sequenced on an illumine NovaSeq 6000 paired‐end 100. Approximately 5000 cells were sequenced to a read depth of approximately 60000 reads per cell. The expression matrix was normalized and visualized with PCA, tSNE, and UMAP, followed by additional analyses using a winseurat and loupe browser. The genes were identified through the databases at https://panglaodb.se/search.html and http://bio‐bigdata.hrbmu.edu.cn/CellMarker/index.html. Specifically, monocytes were defined as cells positive for *Cd14*, *Itgam* (CD11b), *Ccr2*, and *Fcgr3*, and macrophages were defined as cells positive for *Fcgr1*, *Mertk*, and *Adgre1* (F4/80).^[^ ^43^ ^]^

### Liquid Chromatography‐Tandem Mass Spectrometry

Mouse lungs were collected, flash frozen in liquid nitrogen (LN2), and stored at −80 °C. Samples were analyzed using a liquid chromatography‐tandem mass spectrometry system equipped with 1290 HPLC (Agilent), Qtrap 5500 (ABSciex) and reverse phase column (Pursuit 5 C18 150 × 2.1 mm). Mobile phase A was 0.1% acetic acid in H_2_O, and mobile phase B was 0.1% acetic acid in ACN/MeOH (84/16, v/v). The flow rate was 250 µl/min, and the column oven was set at 25 °C. The separation gradient consisted of 35–45% B for 1.25 min, 45–55% B for 2 min, 55–66% B for 5.5 min, 66–72% B for 4 min, 72–82% B for 2.5 min, 82–95% B for 1.5 min, a hold at 95% of B for 1.5 min, 95–35% B for 0.1 min, and a hold at 35% of B for 3.9 min. Multiple reaction monitoring was in negative ion mode, and the extracted ion chromatogram corresponding to the specific transition of each analyte was used for quantification. The calibration range for each analyte was 0.1−10000 × 10^−9^ m (*r* ^2^ ≥ 0.99). Data analysis was performed using Analyst 1.7.1 software.

### Statistics

All data are presented as the mean and SEM. Statistical comparisons between groups were performed using t‐test, 1‐way ANOVA, and 2‐way ANOVA using GraphPad Prism software. Survival curves in groups of trained versus untrained mice following lung injury were compared by log‐rank tests. Statistical significance was defined as a *P*‐value <0.05.

## Conflict of Interest

The authors declare no conflict of interest.

## Author Contributions

Y.‐Y.K. and D.‐Y.K. designed and performed experiments, analyzed the data, and wrote the manuscript; S.‐Y.L., H.‐J.K., and T.K. performed experiments; J.A.C. and T.L. analyzed the data; and E.Y.C. conceived the project, designed the experiments, analyzed the data, and wrote the manuscript.

## Supporting information

Supporting Information

## Data Availability

The data that support the findings of this study are available from the corresponding author upon reasonable request.
