# Supplementary material for: Innate Immune Training Initiates Efferocytosis to Protect against Lung Injury
Source: Adv Sci (Weinh). 2024 Jan 26;11(14):2308978. doi: 10.1002/advs.202308978 (PMC11005705; doi:10.1002/advs.202308978)
Supplement: Supplementary file 1 — Supporting Information [file ADVS-11-2308978-s001.pdf]

ADVANCED SCIENCE
Open Access

Supporting Information

for *Adv. Sci.*, DOI 10.1002/advs.202308978

Innate Immune Training Initiates Efferocytosis to Protect against Lung Injury

*Yoon-Young Kang, Dong-Young Kim, Sang-Yong Lee, Hee-Joong Kim, Taehawn Kim, Jeong A. Cho, Taewon Lee and Eun Young Choi**

Supporting Information (SI)

Innate immune training initiates efferocytosis to protect against lung injury

Yoon-Young Kang[1,2], Dong-Young Kim[1], Sang-Yong Lee[1,2], Hee-Joong Kim[1,2], Taehawn Kim[1], Jeong A Cho[1], Taewon Lee, and Eun Young Choi[1,2,]*

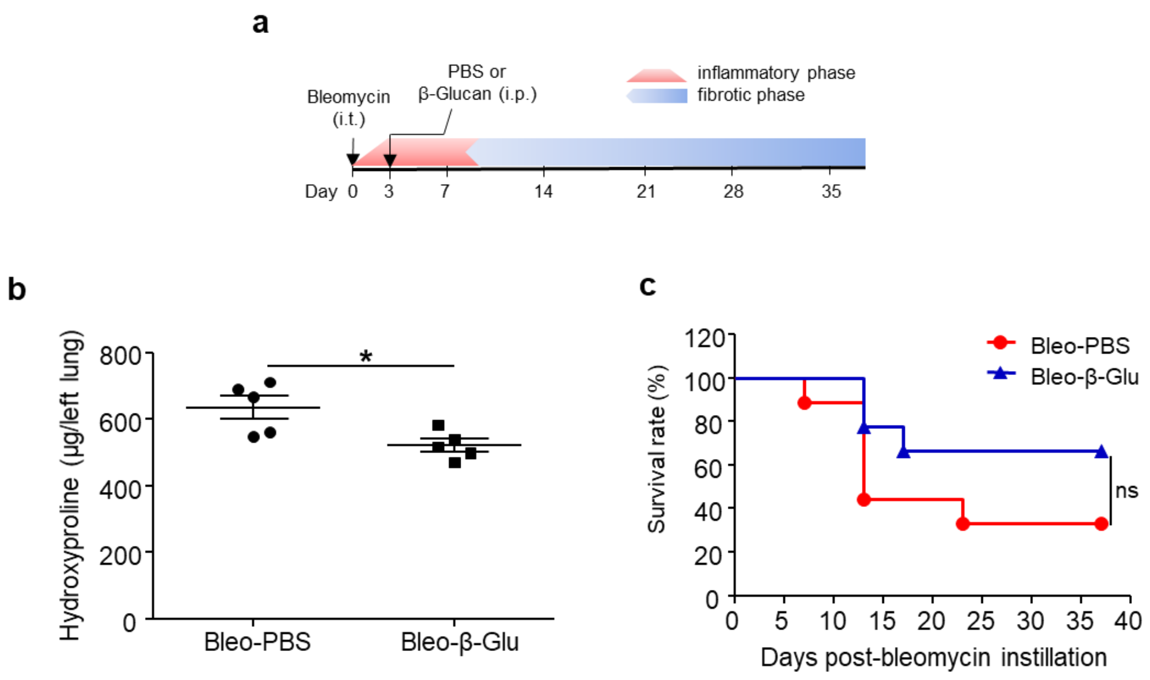

Supplementary Figure 1: Effect of therapeutic administration of β-Glucan on lung injury. **a** Schematic diagram of β-glucan treatment after the initiation of lung injury. Mice were intratracheally instilled with bleomycin, followed by intraperitoneal administration of β-glucan at 3 dpbi, and monitored for 37 days. **b** Hydroxyproline content of lung tissues at 37 dpbi. n = 6 mice per group. *$p < 0.05$ by one-way ANOVA. **c** Survival rate of PBS- or β-glucan-treated mice with bleomycin-induced pulmonary fibrosis. n = 9 mice per group. ns, non-significant by log-rank test.

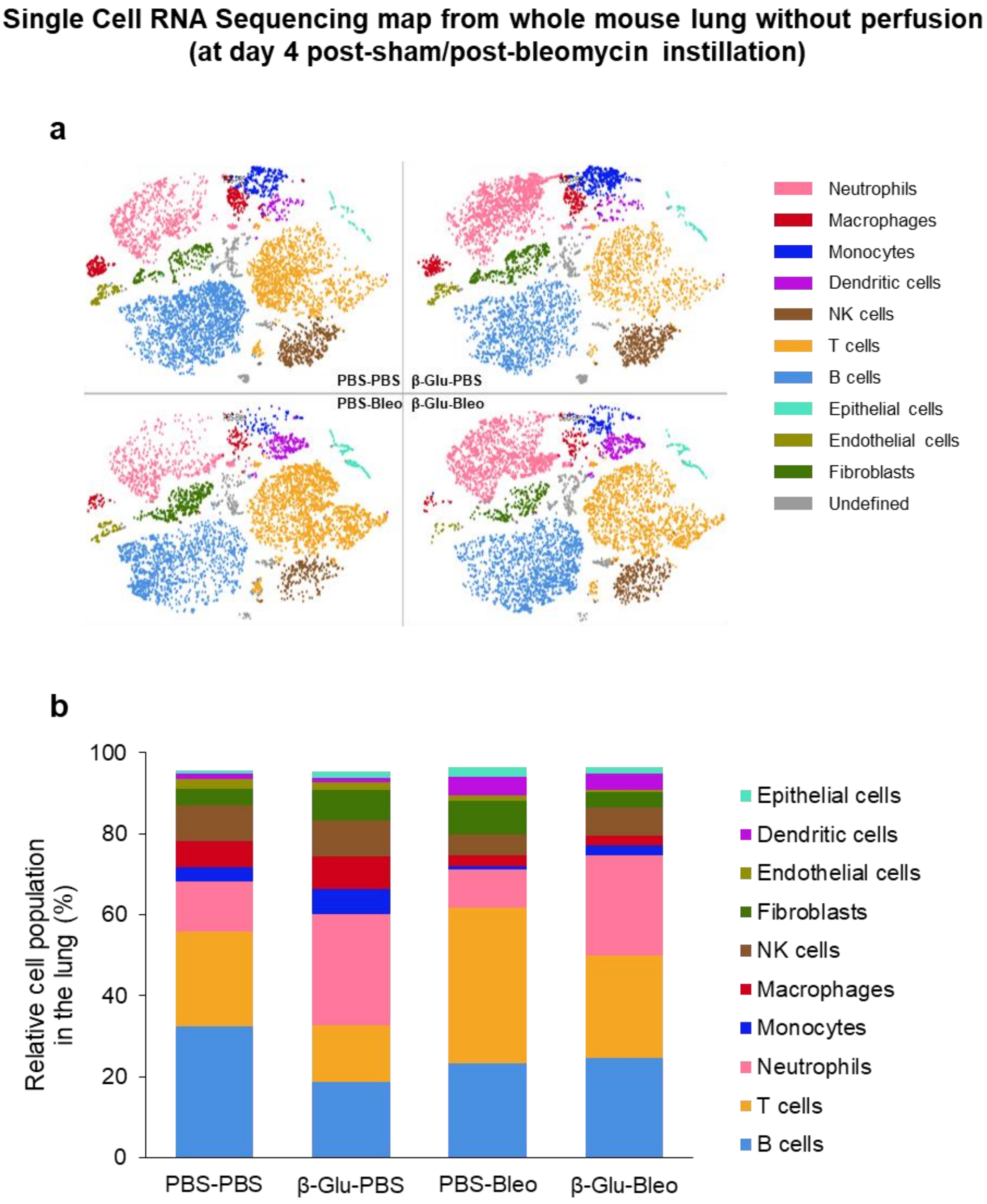

Supplementary Figure 2: Single-cell RNA seq reveals distinct cell compositions in whole lungs of untrained and trained mice before and after injury. a t-Distributed stochastic neighbor embedding (tSNE) plots of immune and non-immune cells (PBS-PBS *n* = 7126 cells, β-Glu-PBS *n* = 6533 cells, PBS-Bleo *n* = 6423 cells, β-Glu-Bleo *n* = 6695 cells/group) in the lungs of untrained and trained mice 4 days after PBS or bleomycin instillation (early inflammation phase). **b** Relative cell composition of whole lungs.

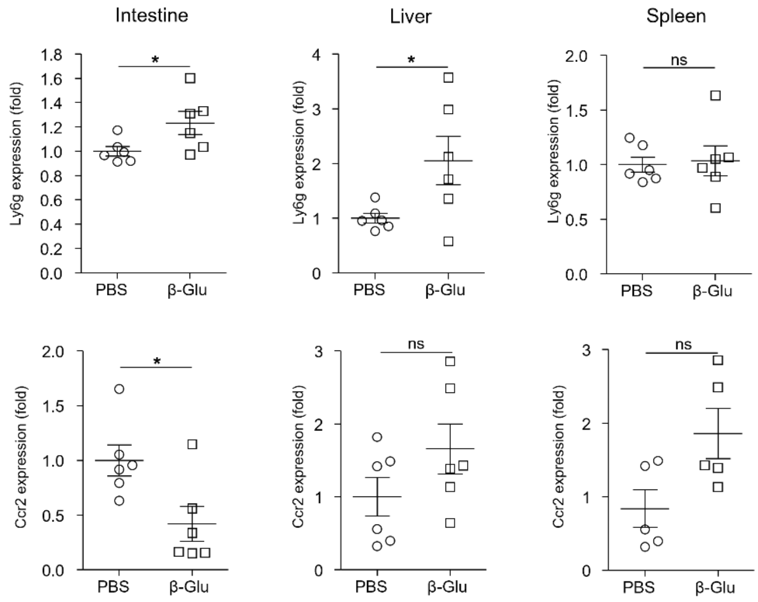

Supplementary Figure 3: Systemic β-glucan-induced trained immunity leads to accumulation of neutrophils in other tissues. Relative level of expression of *Ly6g* (a neutrophil marker) and *Ccr2* (a bone marrow-derived monocytes/macrophage marker) in the intestine, liver, and spleen at day 7 after β-glucan or PBS treatment. *n* = 6 mice per group. **p* < 0.05 by t-tests.

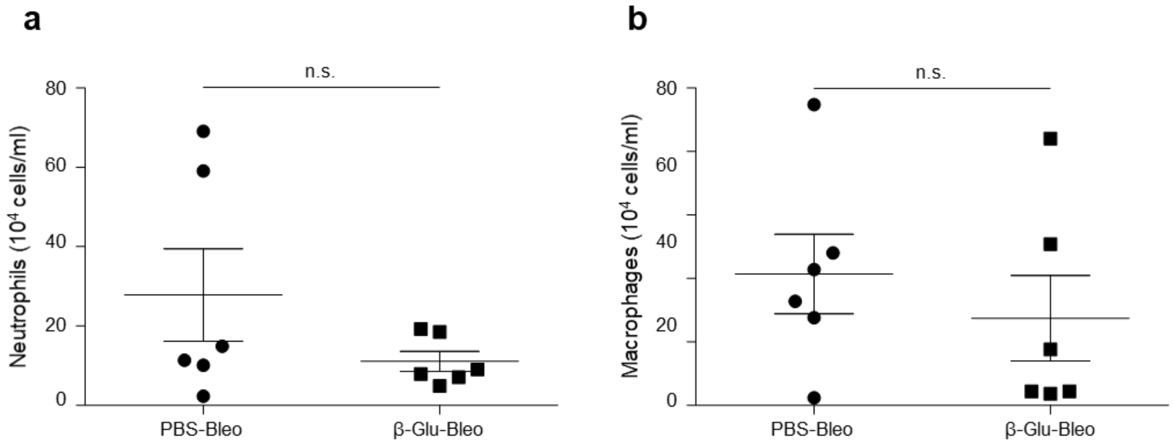

Supplementary Figure 4: Systemic β-glucan-induced trained immunity leads to comparable accumulation of myeloid cells in the lungs of mice following injury. a-b Numbers of accumulated (**a**) neutrophils ($CD11b^+Gr1^+$) and (**b**) macrophages ($F4/80^+CD45^+$) in the BALF of untrained and trained mice 3 days after PBS (sham) or bleomycin instillation. *n* = 6 mice per group. *$p < 0.05$; **$p < 0.01$; ***$p < 0.001$ by t-tests.

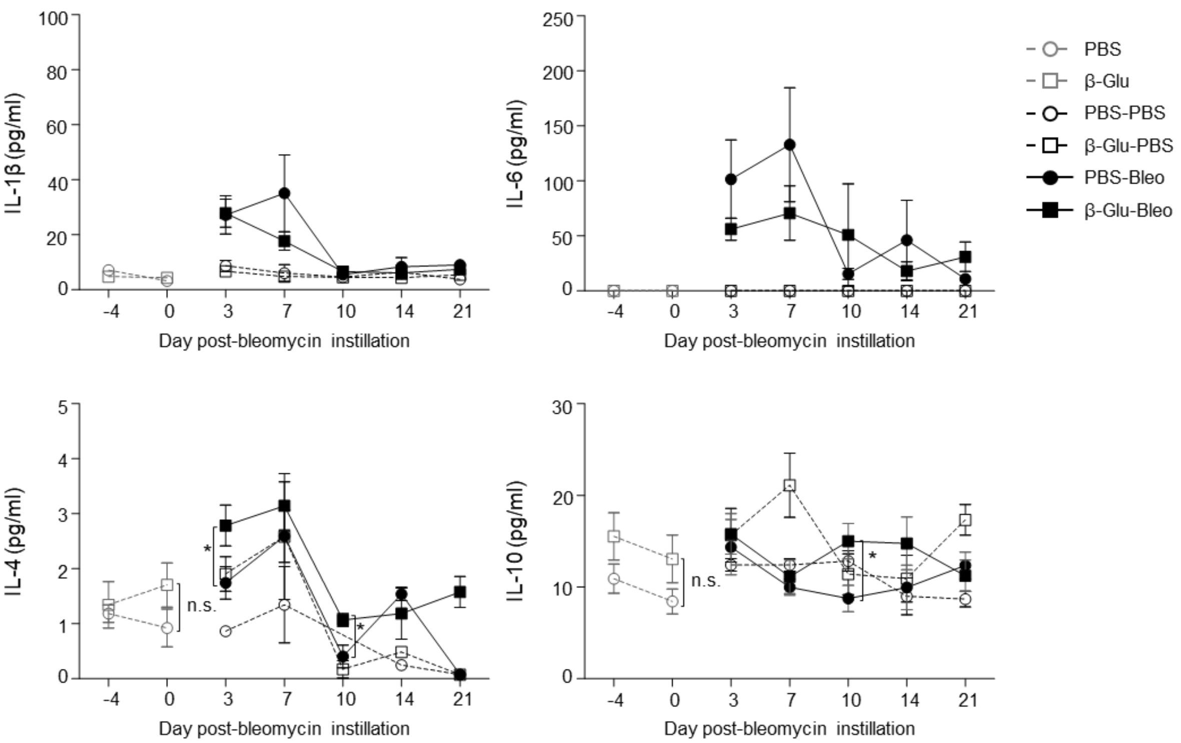

Supplementary Figure 5: Cytokine profiles in the lungs of untrained and trained mice during the course of lung injury. Levels of cytokines in the BALF of untrained and trained mice before and after lung injury. n = 3–6 mice per group. *$p < 0.05$; **$p < 0.01$; ***$p < 0.001$ by two-way ANOVA.

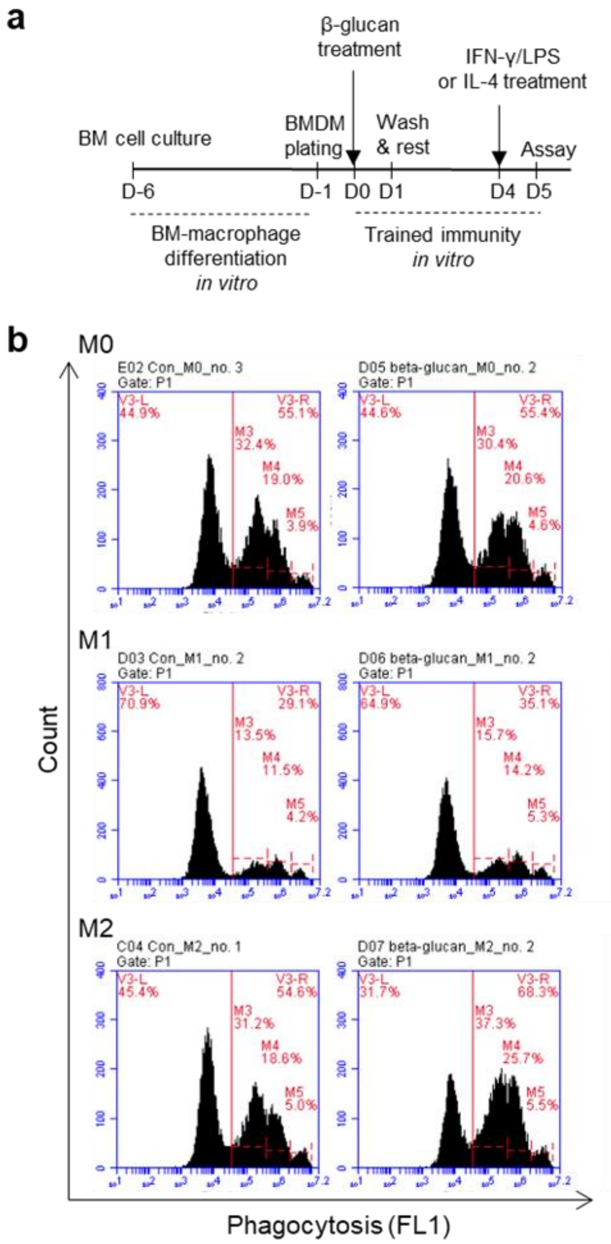

Supplementary Figure 6: Enhanced phagocytosis by *in vitro*-trained BMDMs. a Scheme for *in vitro* training of bone marrow-derived macrophages (BMDM) involving stimulation, allowing M1/M2 polarization. **b** Representative flow cytometry plots showing phagocytosis of phosphatidylserine-coated fluorescent beads by untrained or trained BMDMs incubated in the absence (M0-type) or presence of LPS/IFN-γ (M1-type) or IL-4 (M2-type). Differences in phagocytic ability of the cells depended on fluorescence intensity (M3, low; M4, medium; M5, high).

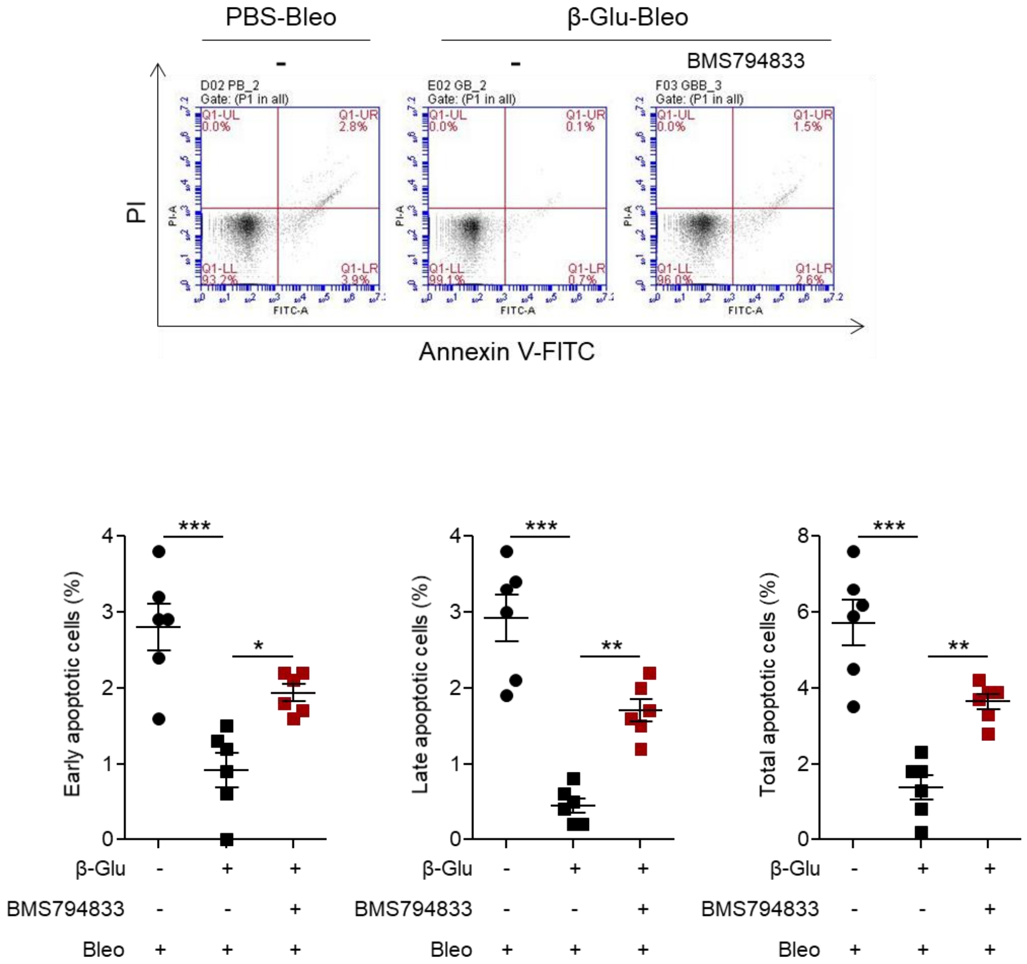

Supplementary Figure 7: Percentage of apoptotic cells in the BALF of untrained and trained mice with lung injury. The mice were trained with β-glucan or not. After 7 days, the mice were intraperitoneally injected with BMS794833, a specific efferocytosis blocker, 30 min prior to bleomycin instillation. BALF was collected at 1 dpbi and the cells were analyzed for the percentage of apoptotic cells using annexin V and propidium iodide. Upper panels are representative plots. *n* = 6 mice per group. *p < 0.05; **p < 0.01; ***p < 0.001 by t-tests.

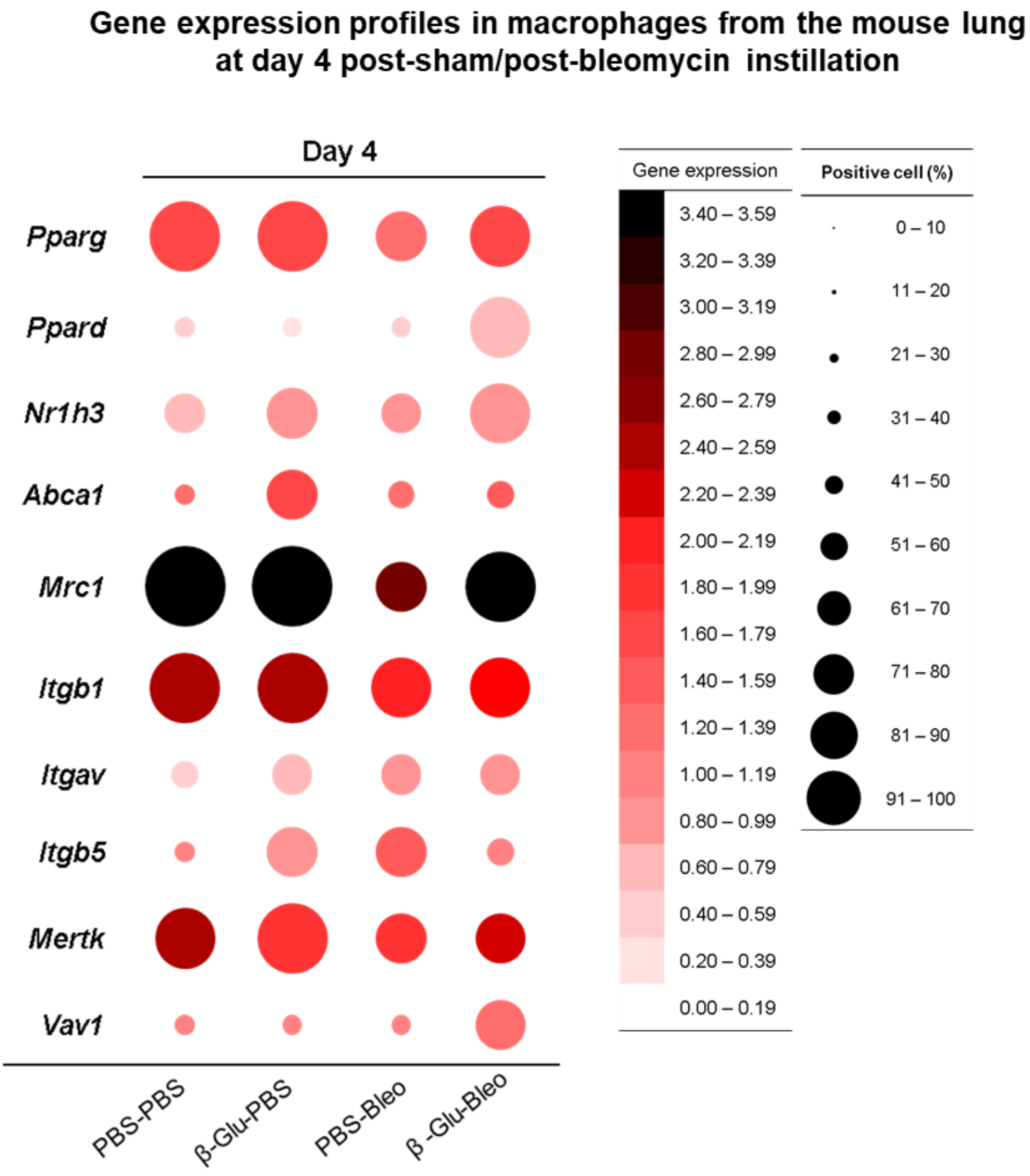

Supplementary Figure 8: Expression of genes associated with efferocytosis in alveolar macrophages of untrained and trained mice during the course of lung injury, based on the results of scRNA sequencing. Lungs of untrained and trained mice were collected 4 days after PBS or bleomycin instillation (early inflammation phase). Expression profiles in macrophages (*Fcgr1*$^+$, *Mertk*$^+$, *Adgre1*$^+$). The dot plots show the percentages of cells (dot size) expressing the indicated genes and their relative levels of expression (color intensity).

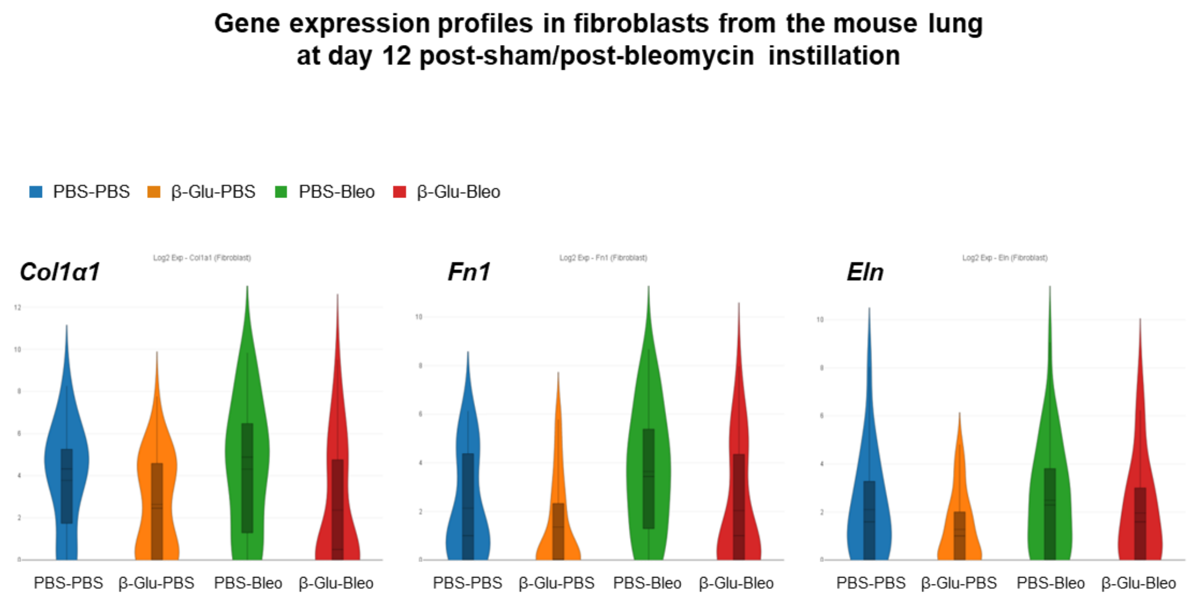

Supplementary Figure 9: Expression of genes commonly used as fibrosis markers in lung fibroblasts of untrained and trained mice before and after injury, based on the results of scRNA sequencing. Violin plots of genes commonly used as fibrosis markers in fibroblasts. Lungs were collected 12 days after PBS or bleomycin instillation (early fibrosis phase).

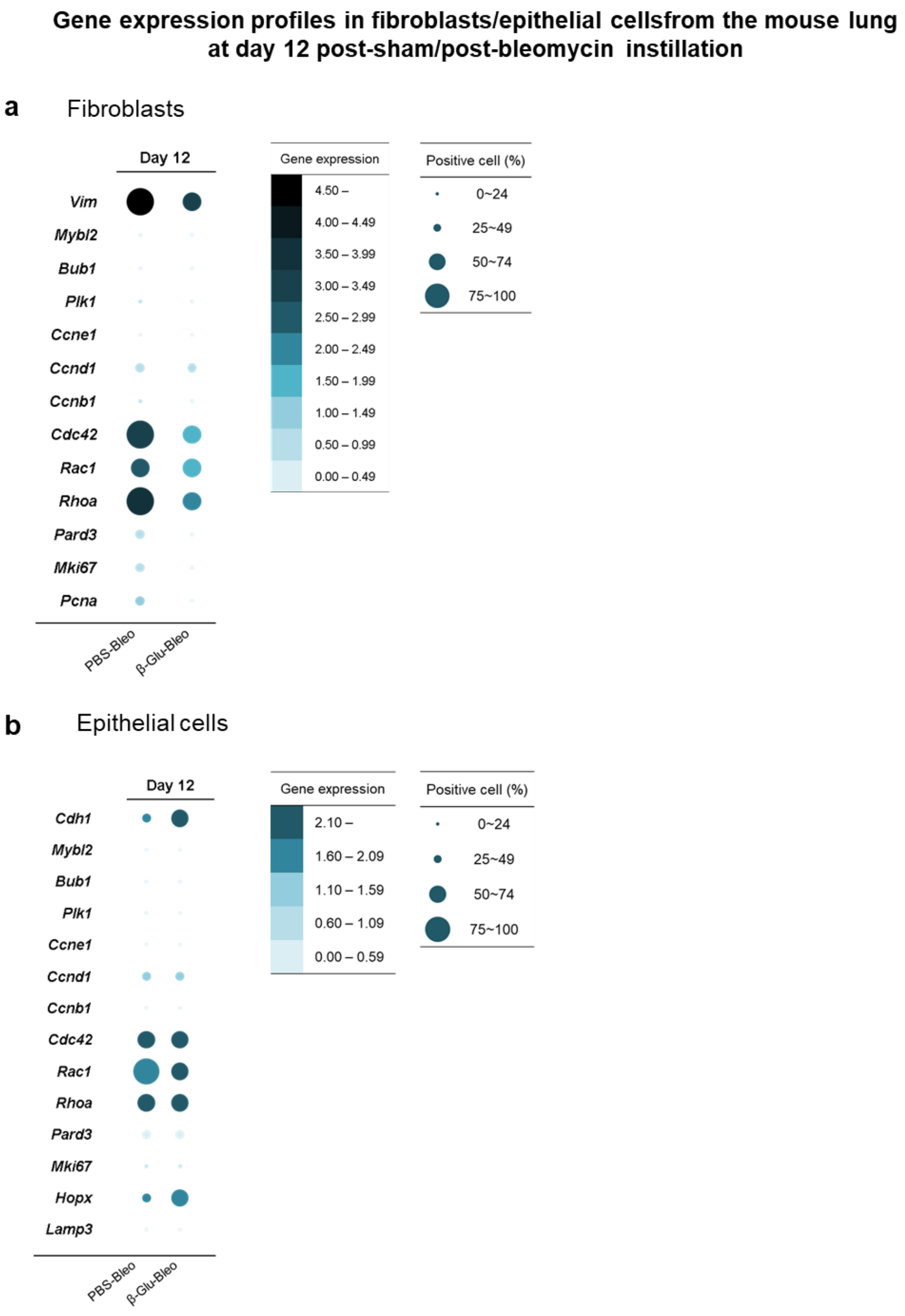

Supplementary Figure 10: Expression of genes associated with cell activation, differentiation, and proliferation in lung fibroblasts and lung epithelial cells of untrained and trained mice after injury, based on the results of scRNA sequencing. a, b Lungs were collected 12 days after bleomycin instillation (the early fibrosis phase), and expression was analyzed in fibroblasts (**a**) and epithelial cells (**b**). The dot plots show the

percentages of cells (dot size) expressing the indicated genes and their relative levels of expression (color intensity).

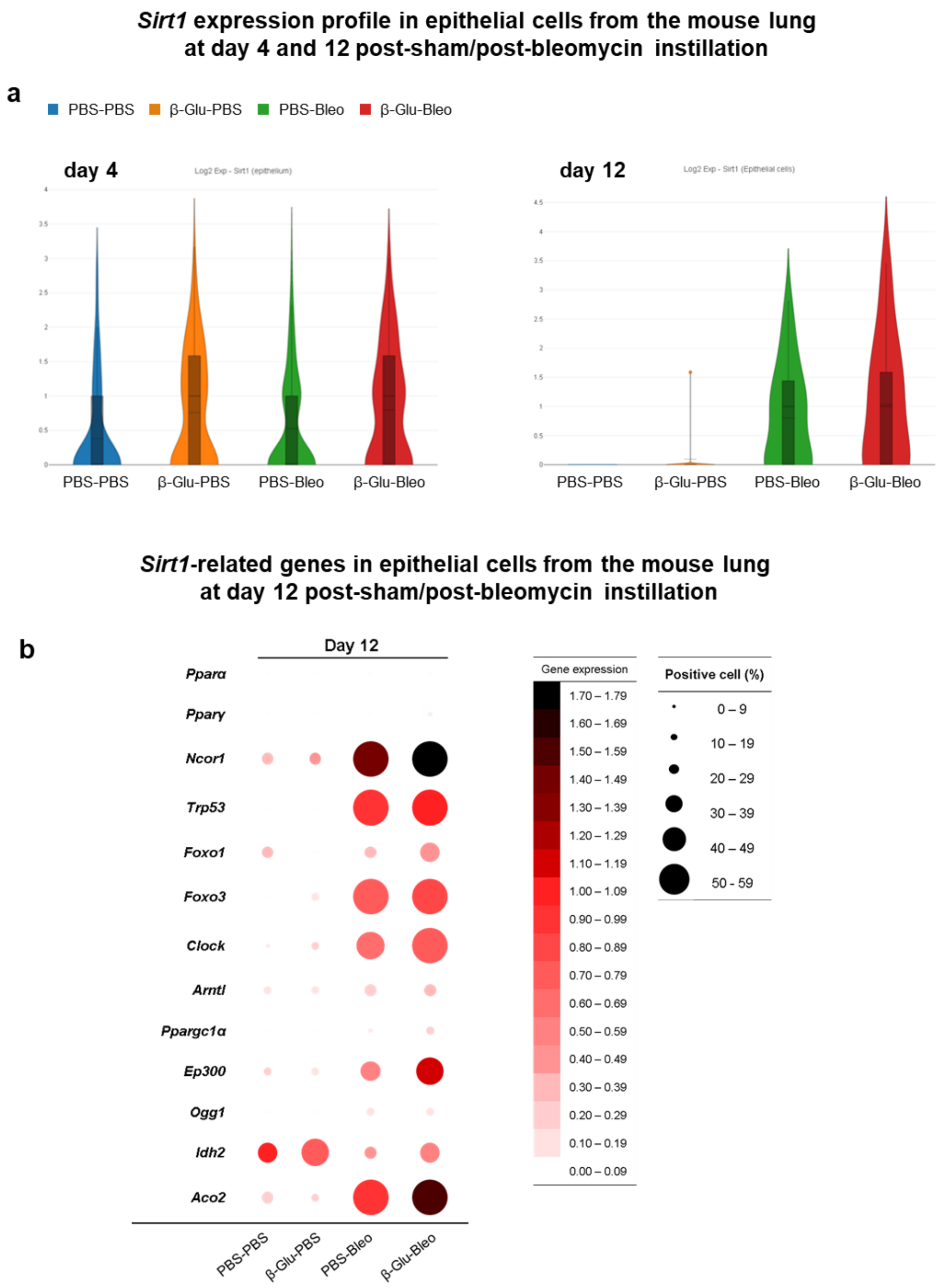

Supplementary Figure 11: Expression of *Sirt1* and *Sirt1*-related genes in lung epithelial cells of untrained and trained mice before and after injury, based on the results of

scRNA sequencing. a Violin plots of *Sirt1* gene expression in lungs collected 4 (early inflammation phase) and 12 (early fibrosis phase) days after PBS or bleomycin instillation. **b** *Sirt1*-related gene expression profiles in epithelial cells of lungs collected 12 days after PBS or bleomycin instillation. The dot plots show the percentage of cells (dot size) expressing indicated genes and their relative levels of expression (color intensity).

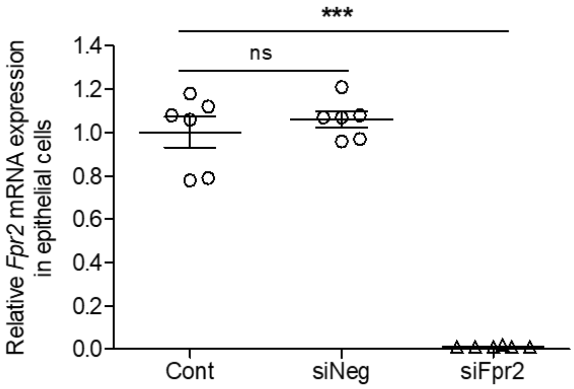

Supplementary Figure 12: Knockdown of the *Fpr2* gene in lung epithelial cells following siRNA transfection. *Fpr2* knockdown was verified by real time PCR. Relative expression was normalized to 18s RNA expression. $n = 5$ per group. ****p* < 0.001 by one-way ANOVA.

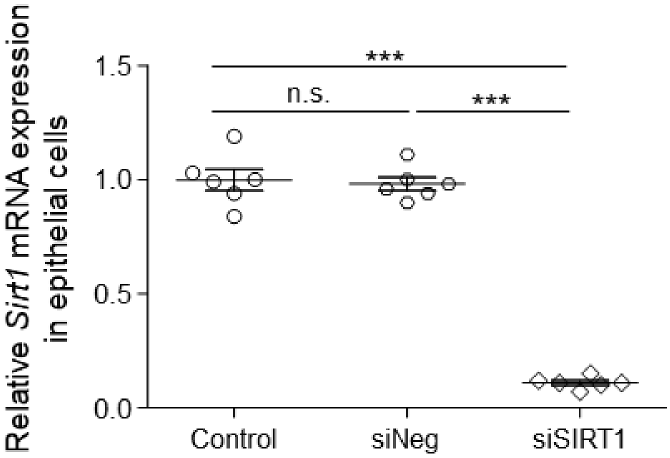

Supplementary Figure 13: Knockdown of the *Sirt1* gene in lung epithelial cells following siRNA transfection. *Sirt1* knockdown was verified by real time PCR. Relative expression was normalized to 18s RNA expression. n = 6 per group. ***p < 0.001 by one-way ANOVA.

a

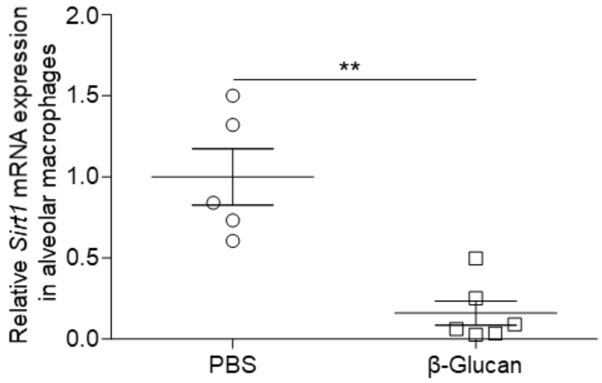

b

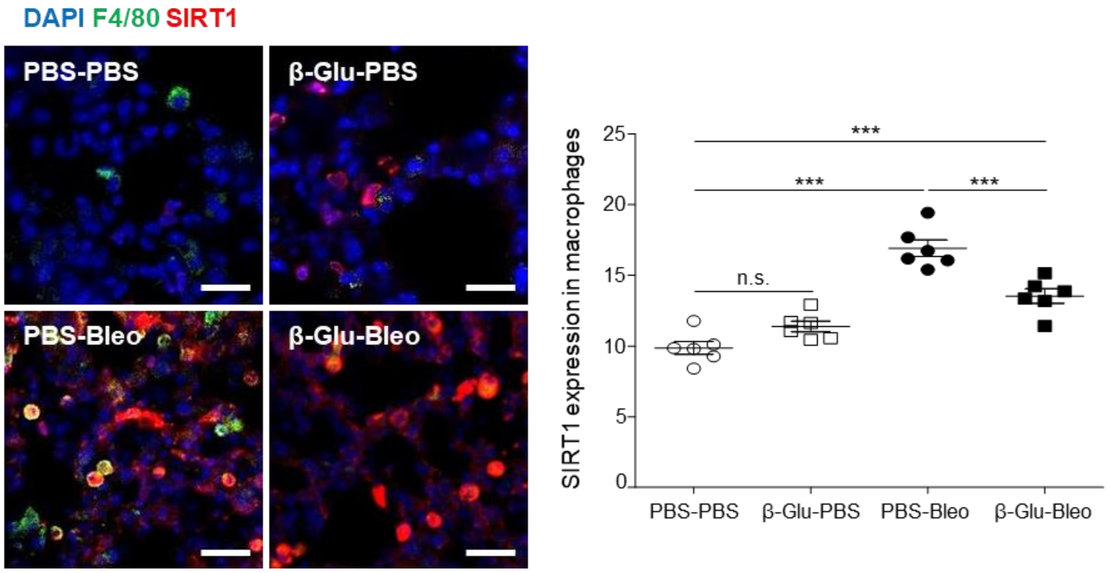

Supplementary Figure 14: Expression of the *Sirt1* gene in alveolar macrophages. a Relative *Sirt1* expression in alveolar macrophages (SiglecF^{+}-sorted cells) isolated from untrained and trained mice 7 days after β-glucan treatment. $n = 5$ per group. $**p < 0.01$ by t-test. **b** Macrophage SIRT1 protein levels in the lungs of untrained and trained mice isolated 7 days after PBS or bleomycin instillation. Scale bar = 20 μm. The right panel shows the quantification of SIRT1^{+}F4/80^{+}-doubly positive cells. $n = 6$ mice per group. $***p < 0.001$ by one-way ANOVA.
